# Polystyrene microplastic stress induces adaptive responses and functional remodeling of gastrointestinal microbiota in fattening lambs

**DOI:** 10.3389/fmicb.2026.1892256

**Published:** 2026-08-05

**Authors:** Mengrong Su, Kaisi Hu, Yingcheng Gao, Liuyan Fang, Bingfeng Zheng, Ruxu Qi, Hongxin Li, Wei Zhang, Wenjie Zhang, Jian Ma

**Affiliations:** 1 College of Coastal Agricultural Sciences, Guangdong Ocean University, Zhanjiang, China; 2 Key Laboratory of Livestock and Poultry Healthy Breeding Technology in Northwest China, Xinjiang Agricultural Vocational and Technical University, Changji, China

**Keywords:** bacterial community, dysbiosis, fattening lamb, gastrointestinal tract, polystyrene microplastic

## Abstract

**Introduction:**

This study aimed to investigate the effects of polystyrene microplastics (PS MPs) on microbial communities in the rumen, jejunum, ileum, cecum and colon of fattening Hu sheep lambs.

**Methods:**

The 16S ribosomal RNA gene amplicon sequencing was employed to analyze the gastrointestinal microbial diversity and composition between the control (CON) and microplastic-exposed (MP, 100 μm PS MPs, 150 mg/d per lamb for 40 d) groups, each with 10 lambs.

**Results:**

Alpha diversity analysis revealed that microplastics exposure slightly reduced the ruminal Shannon index (*p* = 0.087), but significantly increased the Chao1 and PD indexes in the ileum, as well as the Shannon and PD indexes in the colon (*p* < 0.05). At the phylum level, the CON group exhibited a significantly higher Tenericutes abundance than MP group (*p* < 0.05) in the ileum. Compared with CON group, the relative abundance of Proteobacteria was increased by 51.54 and 138.3% in the jejunum and ileum of MP group, respectively (0.05 < *p* < 0.10). Conversely, the relative abundance of Proteobacteria in the cecum (p < 0.05) and colon (*p* = 0.056) of MP group was lower than that in CON group, while the Firmicutes and Elusimicrobia relative abundances in the colon were significantly higher in MP group than CON group (*p* < 0.05). At the genus level, after microplastics exposure, the relative abundance of unclassified Muribaculaceae showed a decreasing trend in the rumen (0.05 < *p* < 0.10). In the jejunum of MP group, the pathogenic genus Ralstonia abundance exhibited a slight increase (0.05 < *p* < 0.10), whereas the Mycoplasma and [Eubacterium] coprostanoligenes group abundances in the ileum were significantly decreased (*p* < 0.05). The relative abundance of Rikenellaceae RC9 gut group was decreased in the cecum (*p* < 0.05) after microplastics exposure.

**Discussion:**

Overall, PS MPs induced segment-specific dysbiosis of the gastrointestinal microbiota across multiple intestinal segments in lambs, and the ileum and colon were the most sensitive to microplastics exposure, with their microbial diversity, dominant phyla and genera, and interspecies interactions all being disrupted.

## 1 Introduction

Plastic products are extensively used owing to the low cost, corrosion resistance, electrical insulation and lightweight properties. The growing demand for plastic products has driven a rapid expansion of the global production chain associated with plastics. According to the statistics, approximately 400 million tonnes of plastics are produced annually, and this figure is projected to more than double by 2050 (Lim, 2021). However, due to the poor degradability of plastics and inadequate recycling practices, vast quantities of plastic waste have accumulated in the environment. Although plastics have brought great convenience to human society, they simultaneously impose substantial risks to both ecological systems and animal health. Plastic waste in the environment forms plastic fragments of different sizes through physical abrasion and chemical aging processes. Among them, microplastics are defined as plastic particles or fragments with a diameter of less than 5 mm (Hartmann et al., 2019). Microplastics are primarily derived from two sources: primary microplastics and secondary microplastics (Mishra et al., 2025). Primary microplastics are small particles intentionally added to consumer and commercial products, such as toothpaste and cosmetics, which can enter the environment directly. Secondary microplastics are plastic fragments generated in the environment through the physical abrasion, photochemical degradation, or biodegradation of macroplastics. The microplastics are widely distributed in environmental matrices including soil, lakes and air, and have emerged as ubiquitous pollutants. Research on the influence of microplastics pollution has become one of the most active research topics across the globe (Shiny et al., 2026). Microplastics have been listed as one of the four major global environmental threats along with global climate change, ocean acidification and ozone depletion (Geng et al., 2021).

Microplastics are characterized by large specific surface area, chemical stability, small size and resistance to degradation. These properties facilitate their rapid transport across different environmental compartments, allowing them to enter food chains, undergo trophic transfer and accumulate in animal tissues (Peng et al., 2020). An increasing number of evidence has demonstrated that microplastics exert multiple toxicological effects on different organisms (Su et al., 2025). Agricultural ecosystems serve as major sinks for environmental microplastics, with livestock-related sources being of particular concern. In agricultural environments, microplastics mainly originate from plastic films used in greenhouse, silage and mulching, as well as plastic pipes and faucets, biological waste products applied as fertilizer (e.g., sewage sludge), contaminated feed and general-purpose plastic equipment (Wu et al., 2021; Ziajahromi et al., 2024). Among these sources, plastic mulching films have been identified as the predominant pathway through which microplastics enter the livestock industry (Ramachandraiah et al., 2022). Furthermore, soil serves as a major reservoir for microplastics, and plant roots can readily absorb microplastic particles and transfer them to edible parts through the xylem pathway (Li et al., 2023). Consequently, during grazing, the animals on the farm are highly likely to ingest microplastics through the grass or surface water (Jin et al., 2022). Ingestion is the primary route by which microplastics enter animal bodies, and besides, inhalation (Lee et al., 2022) and dermal contact (Khan et al., 2024) have also been identified as potential exposure pathways. These microplastics can adversely affect the health and production performance of animals, which have tangible and severe threats to the livestock industry. In poultry production, a previous study has found that microplastics exposure reduced growth performance and antioxidant capacity, caused damage to the intestine, liver, spleen and kidney, and disrupted gut microbial homeostasis and intestinal metabolism of chicken (Li et al., 2023). Microplastics were also found to accumulate in the small intestine, liver, and bones of chickens, with smaller-diameter particles exhibiting higher deposition levels in bone tissue. Also, microplastics can inhibit energy and lipid metabolism in skeletal muscle, induce oxidative stress and potentially exert neurotoxic effects on skeletal muscle (Chen et al., 2023). In ruminants, a study has reported that microplastics exposure of lambs can lead to reduced average daily weight gain and meat quality, abnormal blood metabolism and oxidative stress. Besides, microplastics exposure also caused severe damage to the jejunum and altered the fermentation characteristics and microbial community of the rumen, which negatively affected the digestion and absorption of lambs (Chang et al., 2024).

The gastrointestinal tract is the primary organ for digestion and nutrient absorption of animals, and the gut microbiota plays a pivotal role in maintaining host health (Li et al., 2022; Negash, 2022). Gut homeostasis is sustained through the intricate interactions among the gut microbiota, mucosal immunity, epithelial cells and nutrients (Antonini et al., 2019). The nutrients uptake and immune regulation are both dependent on a stable microbial composition and structure (Kamada et al., 2013). Therefore, maintaining the stability of gut microbiota is critical for the preservation of intestinal homeostasis and health. Nevertheless, the gastrointestinal tracts are the main channel through which livestock and poultry ingest microplastic particles, and the intestinal microbiota are inevitably affected. Many studies have revealed that microplastics can induce gut microbial dysbiosis and compromise intestinal barrier integrity, but the majority of these studies have focused primarily on aquatic organisms and rodents (Liu et al., 2023; Xie et al., 2022; Shi et al., 2025; Kalra et al., 2026). Unlike monogastric animals, the rumen is a unique digestive organ of ruminants, in which microorganisms establish a complex microbial ecosystem that influences multiple physiological functions of the host. Moreover, the rumen environment of ruminants may exacerbate the physical fragmentation effects of microplastics, thereby not only affecting the microbial communities within the rumen but also potentially exerting adverse effects on the microbiota of the hindgut. At present, the information of the influence of microplastics on the community structure in the gastrointestinal tracts of ruminants remains scarce. Accordingly, this study aimed to investigate the effects of polystyrene microplastics on the microbial communities in the rumen, jejunum, ileum, cecum and colon of fattening Hu sheep lambs, with the objective of elucidating the potential hazards of microplastics to multiple gastrointestinal segments of lambs. The core innovations of this study are as follows: first, it systematically reveals the differential effects of microplastics on microbial communities across multiple intestinal segments in the ruminant digestive tract; second, it explores the cross-regional cascade effects of rumen microbiota disturbance on hindgut microecology, which provide critical scientific evidence for assessing the ecological and health risks of microplastics in ruminant production.

## 2 Materials and methods

### 2.1 Animals and experimental design

The polystyrene microplastics (PS MPs) were purchased from Tesulang Chemical Materials Co. Ltd. (Dongguan, China), with a particle size of approximately 100 μm. This study was conducted at a commercial sheep farm located in Changji (Xinjiang, China), which has approximately 10,000 ewes. A total of 20 male Hu sheep lambs with similar age (90 ± 5 d) and body weight (25.76 ± 0.38 kg) were selected as experimental animals. All lambs were marked with ear tags and randomly assigned to two groups of 10 lambs each: a control group (CON) and a microplastic-exposed group (MP). During the experimental period, the lambs of CON group received starch capsules orally once daily, and each lamb from MP group received 150 mg/d of 100 μm PS MPs encapsulated in starch capsules. The dosage was selected based on the previous studies (Chang et al., 2024; Choi et al., 2021). The length of adaptation period was 7 d, and the formal experimental period lasted for 40 d. All experimental animals were housed individually in a separate pen (1.5 m × 2 m). Experimental diet was formulated according to the nutrient requirements from the Agricultural Industry Recommended Standard of China (NY/T 816–2021), and the basal diet was offered twice daily at 09:00 and 17:00, allowing approximately 5% orts with *ad libitum* access to clean water. Dietary composition and nutritional levels of basal diet are presented in Supplementary Table S1.

### 2.2 Sample collection

On d 41 of the experiment, eight lambs of each group that were close to the group average body weight were selected for slaughter. After a 12 h fasting period, the lambs were stunned by captive bolt stunning and subsequently exsanguinated humanely. The slaughter procedures were conducted in accordance with the national standard operating procedures (GB/T 43562–2023, Sheep Slaughtering, China). After slaughter, the abdominal cavity of lambs was opened, and then the rumen, jejunum, ileum, cecum and colon were carefully isolated and ligated with nylon cords to prevent reflux of digesta from adjacent segments. Digesta samples from each gastrointestinal segment were collected and individually placed into sterile centrifuge tubes. The samples were immediately snap-frozen in liquid nitrogen and subsequently stored at −80 °C until further analysis. From the 16 slaughtered lambs, six lambs (n = 6) were randomly selected from each group for subsequent total DNA extraction, and a total of 60 gastrointestinal digesta samples were obtained.

### 2.3 Microbial DNA isolation

All digesta samples were thawed at 4 °C, and 200 mg of digesta from each gastrointestinal segment was weighed for total DNA extraction using the ZymoBIOMICS DNA Microprep Kit (D4301; Zymo Research, Irvine, CA, USA). This kit employs an optimized lysis buffer system, which can efficiently and accurately extract high-purity DNA from trace samples. The specific procedure for microbial DNA extraction was performed strictly in accordance with the manufacturer’s instructions. The integrity of genomic DNA was assessed by 0.8% agarose gel electrophoresis, and DNA concentration was quantified using a Tecan F200 microplate reader with the PicoGreen fluorescence assay. Finally, the extracted DNA was diluted to 10 ng/μL with sterile ultrapure water and stored at −80 °C for subsequent downstream analysis.

### 2.4 PCR amplification

The V4 region of 16S rRNA gene was amplified from all DNA samples using universal primers 515F (5′-GTGYCAGCMGCCGCGGTAA-3′) and 806R (5′-GGACTACHVGGGTWTCTAAT-3′), which contain a 12-nt unique barcode (Caporaso et al., 2011). PCR amplification was performed in a 50 μL reaction mixture containing 5 μL of 1 × PCR buffer, 5 μL of 0.2 mM dNTPs, 3 μL of 1.5 mM MgSO₄, 1.5 μL of each primer (0.3 μM), 1 μL of TOYOBO KOD-Plus-Neo DNA Polymerase (1 U; KOD-401B) and 2 μL of template DNA (20 ng), with the volume adjusted to 50 μL using sterile ultrapure water. Amplification was carried out using an Applied Biosystems Gene Amp PCR System 9,700 (Thermo Scientific, Waltham, MA, USA) under the following thermal cycling conditions: initial denaturation at 94 °C for 1 min (1 cycle); followed by 30 cycles of denaturation at 94 °C for 20 s, annealing at 54 °C for 30 s, and extension at 72 °C for 30 s; with a final extension at 72 °C for 5 min. Each sample was amplified in triplicate, and the PCR products from exponential phase were pooled in equal volumes for subsequent library preparation. The pooled PCR products were mixed with 6 × loading buffer and loaded onto a 2% agarose gel for target fragment verification. Qualified bands were excised and recovered using the Zymoclean Gel Recovery Kit (D4008; Zymo Research, Irvine, CA, USA), and DNA concentration was quantified using a Qubit 2.0 Fluorometer (Thermo Scientific, Waltham, MA, USA). Finally, the PCR amplicons were pooled at equimolar concentrations for downstream sequencing analysis.

### 2.5 High-throughput sequencing and sequencing data analysis

Sequencing libraries were constructed using the NEBNext Ultra II DNA Library Prep Kit for Illumina (NEB #E7645L; New England BioLabs, Ipswich, MA, USA). Sequencing was performed on an Illumina NovaSeq 6,000 platform using the NovaSeq 6,000 SP Reagent Kit V1.5 (Illumina, San Diego, CA, USA) with a 2 × 250 bp paired-end (PE) sequencing mode. Raw paired-end reads were merged using FLASH (v1.2.11). Reads were demultiplexed and barcodes were removed from the raw reads using sabre based on the unique barcode sequences. Quality control was performed using QIIME2 (v2020.2) with the following filtering criteria: (1) sequences with a mean quality score below 30 were discarded; (2) sequences shorter than 200 bp were removed; and (3) sequences containing any ambiguous bases (N) were excluded. Based on the QIIME2 (v2020.2) platform, raw sequences were denoised and chimeras were removed using the Deblur algorithm to obtain an amplicon sequence variant (ASV) feature table along with their corresponding representative sequences. Subsequently, taxonomic assignment of the ASV representative sequences was performed using a Naïve Bayes classifier trained against the SILVA reference database (v138). A multiple sequence alignment of the representative sequences was then conducted using QIIME2 (v2020.2), and a phylogenetic tree was constructed using the built-in FastTree plugin. To account for differences in sequencing depth across samples, all samples were rarefied to the minimum sequencing depth among all samples, thereby minimizing the potential confounding effect of sequencing depth on community diversity estimates.

Downstream statistical analysis were performed using R software (v4.0.5). Phylogenetic diversity (PD) was calculated using the picante package, while other *α*- and *β*-diversity metrics were computed using the vegan package. The Wilcoxon rank-sum test for pairwise comparisons in α-diversity analysis was performed using the wilcox test function in the stats package, and the Kruskal–Wallis rank-sum test for comparisons between the two groups was performed using the kruskal test function. Beta diversity was visualized using principal coordinates analysis (PCoA) based on Bray–Curtis dissimilarities. To test for differences in microbial community composition between the CON and MP groups, analysis of similarities (ANOSIM) and permutational multivariate analysis of variance (PERMANOVA) were performed using the anosim and adonis functions in the R package vegan, respectively, based on the same distance matrices. Heatmaps were generated after row-wise z-score normalization (z-value = [observed relative abundance − mean relative abundance of the same genus] / standard deviation), which was used to identify dominant genera and perform clustering analysis at the genus level. Linear discriminant analysis effect size (LEfSe) analysis was performed to identify bacterial taxa that show significant differences between the CON and MP groups. In this study, taxa with a linear discriminant analysis (LDA) score > 3.0 were considered to have substantial contributions to the differences between groups.

### 2.6 PICRUST2 prediction

In this study, 16S rRNA gene sequencing data were used as input for Phylogenetic Investigation of Communities by Reconstruction of Unobserved States 2 (PICRUSt2) to predict the functional molecular profiles of each sample. This method infers corresponding KEGG Orthology (KO) metabolic pathways from the KEGG database based on the phylogenetic placement of each amplicon sequence variant (ASV) and abundance information corrected for 16S rRNA gene copy number. The predicted functional profiles can be summarized and analyzed at three hierarchical levels of KO metabolic pathways. Similarly, z-score normalization was applied to the functional features to generate heatmaps for analysis at the third level, and clustering analysis was performed at this level.

### 2.7 Statistical analysis

The statistical analysis were performed using SPSS software (v26.0 for Windows; SPSS Inc., Chicago, IL, USA). Firstly, all data were tested for normality and homogeneity of variance. Independent-samples t-tests were used to compare *α*-diversity indexes and bacterial relative abundances between the CON and MP groups. When data did not meet the assumptions of normality or homogeneity of variance, the non-parametric tests were applied accordingly. Data are presented as means and standard error of the mean (SEM). Statistical significance was set at *p* < 0.05, and trend was considered at 0.05 < *p* < 0.10.

## 3 Results

### 3.1 Data acquisition and analysis

**Figure 1:**
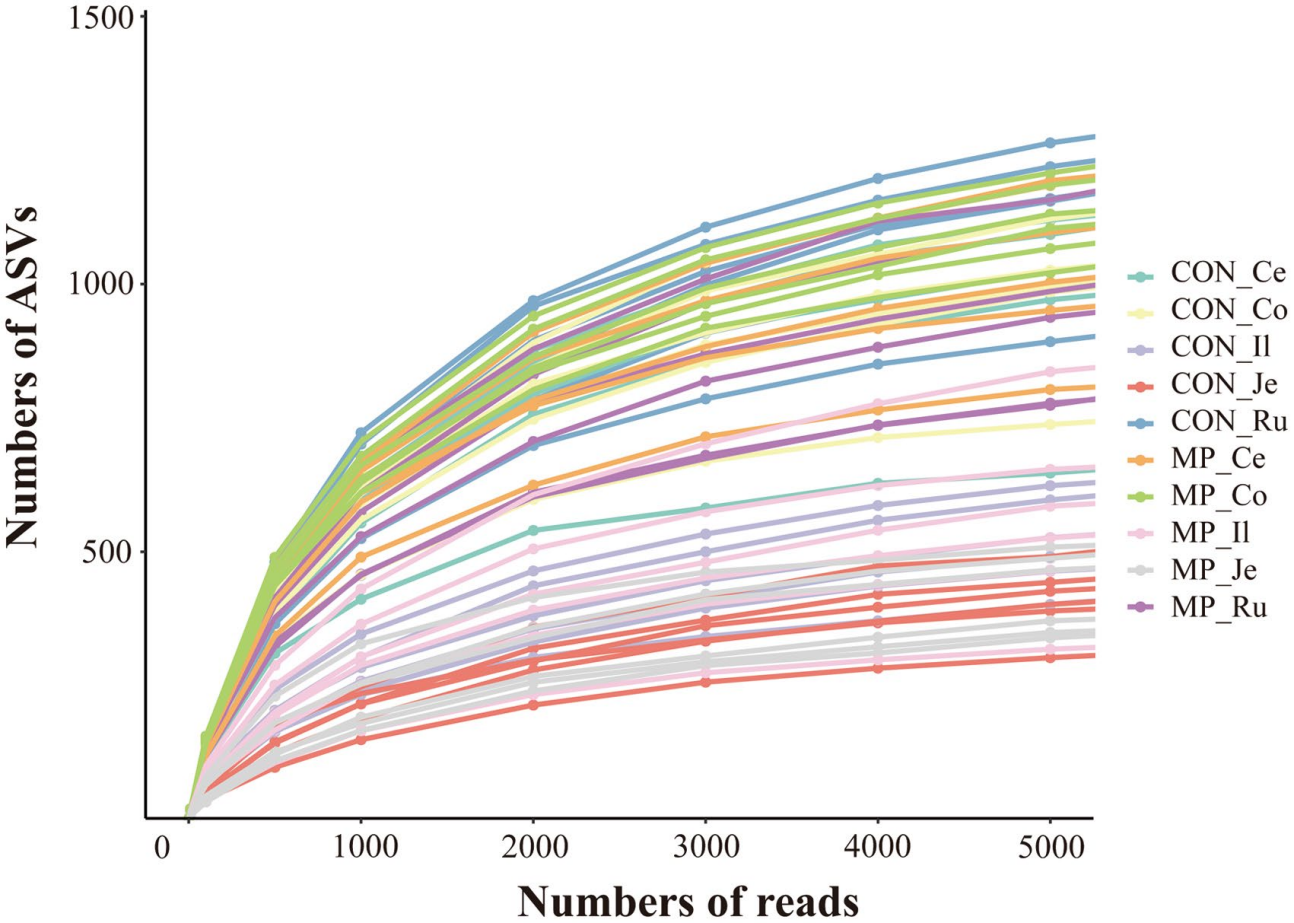
Rarefaction curves based on ASVs of all gastrointestinal digesta samples (*n* = 60). CON, control group lambs (*n* = 6); MP, microplastic-exposed lambs (*n* = 6); Ru, rumen; Je, jejunum; Il, ileum; Ce, cecum; Co, colon.

In this study, a total of 60 digesta samples were collected from the rumen, jejunum, ileum, cecum and colon of lambs. A total of 3,878,917 raw sequences were obtained, with an average of 64,649 ± 1,133 sequences per sample (mean ± standard error) by 16S rRNA gene sequencing (Supplementary Table S2). After quality control procedures (including denoising, filtering and trimming), 3,874,727 high-quality sequences were retained, with an average of 64,579 ± 1,131 sequences per sample. The mean sequence length across all samples was 291.8 ± 0.1 bp (Supplementary Table S2). After denoising the high-quality reads using the QIIME2 pipeline, 9,803, 8,865, 8,117, 8,986 and 9,790 amplicon sequence variants (ASVs) were identified in the rumen, jejunum, ileum, cecum and colon, respectively (Supplementary Table S2). To assess whether the sequencing depth provided sufficient ASV coverage to accurately characterize the bacterial communities in each gastrointestinal segment, rarefaction curves (Figure 1) and Good’s coverage estimates (Q30) (Supplementary Table S2) were generated for each sample. All rarefaction curves reached a plateau, indicating that sufficient sequences were obtained to adequately capture the bacterial diversity in the rumen, jejunum, ileum, cecum and colon. Venn diagram analysis was performed to identify shared and unique ASVs between the two groups across each gastrointestinal segment. Comparison of the shared ASV ratios between the CON and MP groups across the five gastrointestinal segments revealed the following descending order: rumen (93.01%) (Supplementary Figure S1A) > ileum (92.34%) (Supplementary Figure S1C) > cecum (91.21%) (Supplementary Figure S1D) > colon (91.07%) (Supplementary Figure S1E) > jejunum (90.41%) (Supplementary Figure S1B).

### 3.2 Analysis of *α*-diversity indexes of gastrointestinal microbiota across different segments

The α-diversity analysis revealed that Observed species, Chao1, Shannon and PD indexes in the jejunum and cecum were similar between the CON and MP groups (*p* > 0.05) (Table 1). Compared with CON group, the Shannon index in the rumen of MP group was slightly reduced by 5.10% (*p* = 0.087), whereas no significant differences were observed in the other α-diversity indexes (*p* > 0.05). In the ileum and colon, the PD index of MP group was higher than that of CON group (*p* < 0.05). Also, the Shannon index in the colon and the Chao1 index in the ileum were higher in MP group than CON group (*p* < 0.05). Additionally, compared with CON group, the Observed species index was increased by 52.94 and 7.00% in the ileum and colon of MP group, respectively, as well as the Chao1 index in the colon of MP group was increased by 19.82% (0.05 < *p* < 0.10). The Shannon index in the ileum did not differ significantly between the two groups (*p* > 0.05) (Table 1).

**Table 1:** Comparison of *α*-diversity indexes across different gastrointestinal segments in lambs.

Regions	Indexes	CON	MP	SEM	*p*-value
Rumen	Observed species	961.83	830.83	79.485	0.130
	Chao1	976.85	856.05	82.537	0.174
	Shannon	5.59	5.31	0.150	0.087
	PD	73.60	68.23	4.395	0.249
Jejunum	Observed species	708.17	769.33	121.168	0.625
	Chao1	749.08	798.64	128.979	0.709
	Shannon	3.02	3.11	0.450	0.844
	PD	78.59	85.76	11.179	0.536
Ileum	Observed species	534.83	818.00	127.644	0.051
	Chao1	557.97	849.92	129.890	0.048
	Shannon	3.71	3.72	0.411	0.987
	PD	55.65	97.47	13.442	0.022
Cecum	Observed species	723.50	774.17	72.912	0.503
	Chao1	732.46	782.32	74.672	0.519
	Shannon	5.33	5.50	0.174	0.350
	PD	50.54	53.09	3.091	0.429
Colon	Observed species	744.83	886.83	72.019	0.077
	Chao1	752.46	901.61	75.260	0.076
	Shannon	5.32	5.66	0.115	0.014
	PD	47.90	55.90	3.226	0.033

CON, control group lambs (*n* = 6); MP, microplastic-exposed lambs (*n* = 6); SEM, standard error of the mean.

### 3.3 Analysis of β-diversity indexes of gastrointestinal microbiota across different segments

**Figure 2:**
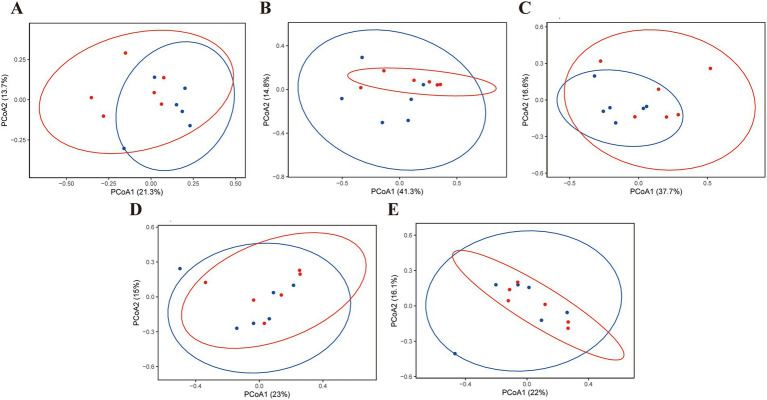
Principal coordinate analysis (PCoA) of bacterial communities in the rumen (*R2* = 0.13, *p* = 0.037) **(A)**, jejunum (*R2* = 0.12, *p* = 0.190) **(B)**, ileum (*R2* = 0.18, *p* = 0.035) **(C)**, cecum (*R2* = 0.10, *p* = 0.276) **(D)** and colon (*R2* = 0.12, *p* = 0.086) **(E)** based on Bray–Curtis dissimilarity distances. CON group (blue) (*n* = 6); MP group (red) (*n* = 6). CON group, control group lambs; MP group, microplastic-exposed lambs.

In the present study, based on the Bray–Curtis dissimilarity matrix, PCoA was performed to compare the gastrointestinal microbial community structures between the CON and MP groups. The bacterial communities in the rumen, jejunum, ileum, cecum and colon did not completely separate from each other (Figure 2). Furthermore, PERMANOVA was conducted to determine whether significant differences in inter-group distances existed. The results revealed significant differences between the two groups in the rumen (R^2^ = 0.13, *p* = 0.037) and ileum (R^2^ = 0.18, *p* = 0.035). Also, a significant trend toward separation was observed in the colon (R^2^ = 0.12, *p* = 0.086), whereas no significant differences were detected in the jejunum (R^2^ = 0.12, *p* = 0.190) and cecum (R^2^ = 0.10, *p* = 0.276) between the two groups (Supplementary Table S3).

### 3.4 Differences of bacterial communities in the rumen

**Figure 3:**
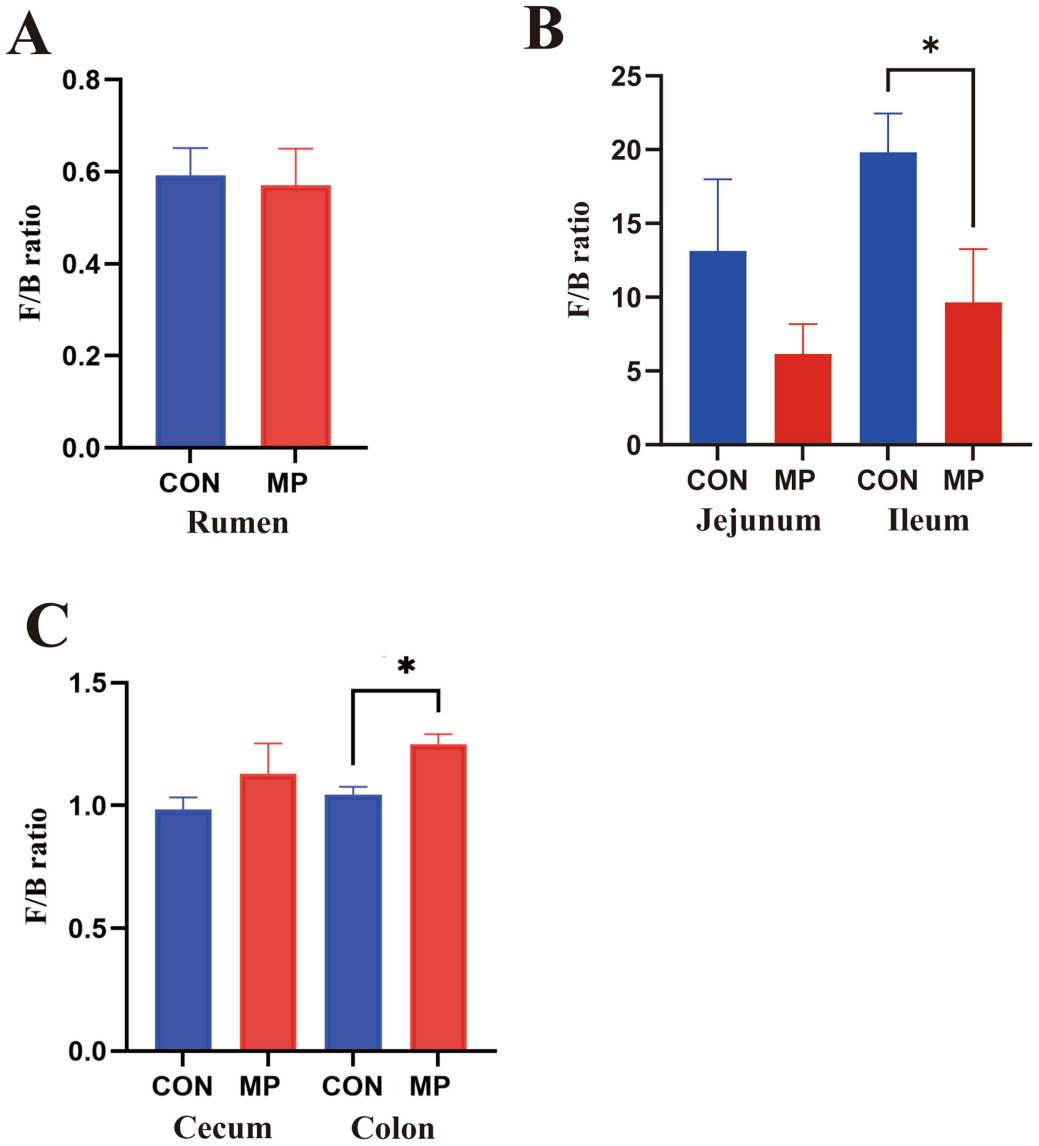
Comparison of the Firmicutes-to-Bacteroidetes (F/B) ratio in the rumen **(A)** small intestine **(B)** and large intestine **(C)** between the CON (*n* = 6, blue bars) and MP groups (*n* = 6, red bars). Asterisks indicate significant differences between the CON and MP groups (*p < 0.05). CON group, control group lambs; MP group, microplastic-exposed lambs.

At the phylum level, a total of 35 phyla were identified from the 12 ruminal digesta samples, with 27 phyla detected in both the CON and MP groups. Bacteroidetes (48.71% in CON vs. 48.82% in MP) and Firmicutes (28.37% in CON vs. 26.89% in MP) were the dominant bacterial phyla in the rumen of lambs, accounting for over 75% of the total relative abundance. Based on the criterion that at least one sample within each group had a relative abundance ≥ 0.5%, a total of 10 predominant phyla were screened in the rumen (Table 2). Among these 10 predominant phyla, 9 phyla showed no significant differences between the two groups (*p* > 0.05), whereas one unclassified phylum was higher in CON group than that in MP group (*p* < 0.05) (Table 2). Moreover, the Firmicutes-to-Bacteroidetes (F/B) ratio did not differ significantly between the two groups (*p* > 0.05) (Figure 3A).

**Table 2:** Comparison of relative abundances (%) of representative bacterial phyla in the rumen between the CON and MP groups.

Items	CON	MP	SEM	*p*-value
Bacteroidetes	48.71	48.82	3.543	0.976
Firmicutes	28.37	26.89	3.357	0.668
Fibrobacteres	8.12	9.95	1.565	0.267
Spirochaetes	7.68	8.39	1.600	0.670
Proteobacteria	3.03	2.71	0.829	0.702
Kiritimatiellaeota	0.64	0.57	0.294	0.835
Tenericutes	0.61	0.51	0.110	0.387
Actinobacteria	0.46	0.51	0.039	0.196
Synergistetes	0.34	0.20	0.318	0.658
Unclassified phylum	0.32	0.04	0.079	0.015

CON, control group lambs (*n* = 6); MP, microplastic-exposed lambs (*n* = 6); SEM, standard error of the mean.

**Figure 4:**
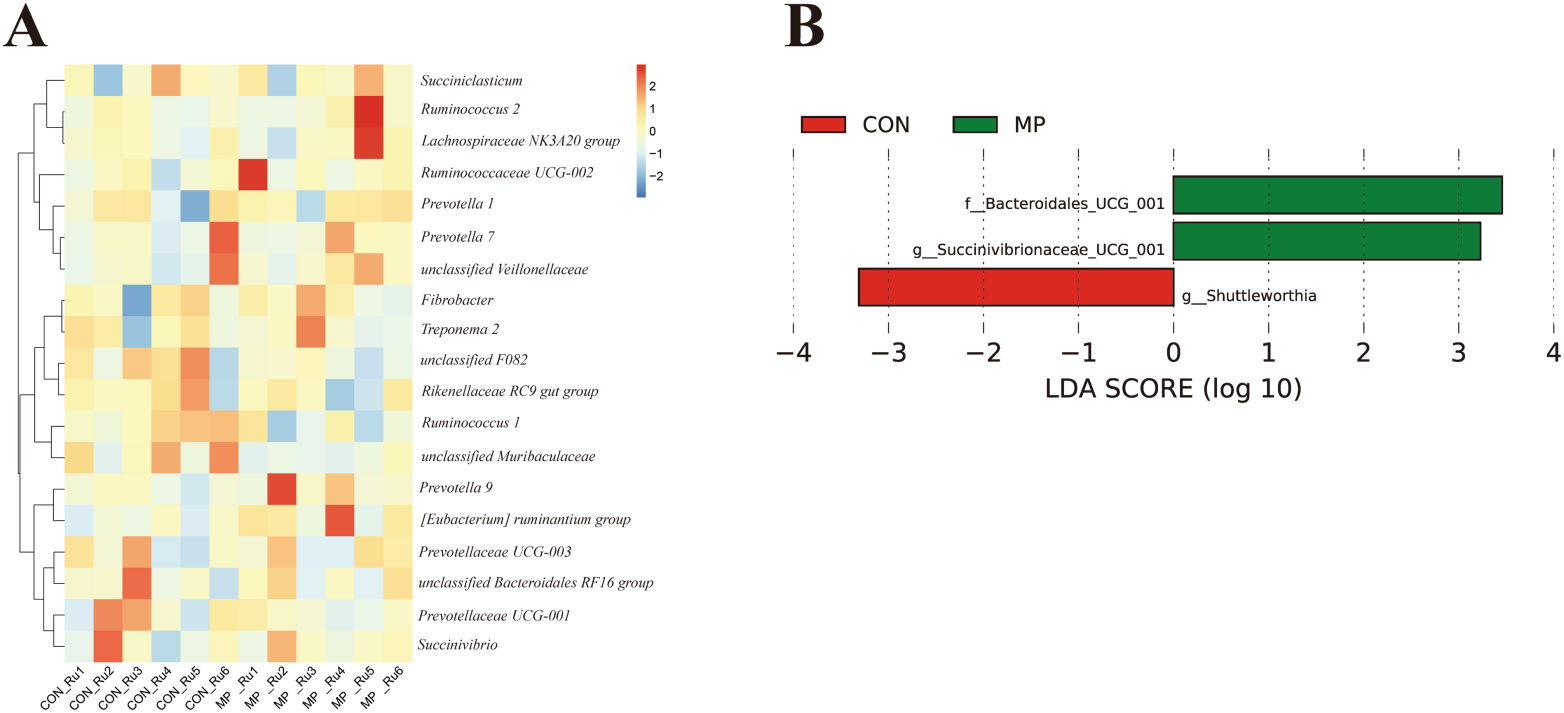
Taxonomic composition of rumen bacteria in the CON (*n* = 6) and MP (*n* = 6) groups. **(A)** Heatmap showing the relative abundances of predominant bacterial genera at the genus level in the rumen. Genera with a relative abundance ≥ 2.5% in at least one sample within a group were included for display. **(B)** Linear discriminant analysis effect size (LEfSe) analysis identified the most differentially abundant genera between the CON (red) and MP (green) groups in the rumen. Genera with a linear discriminant analysis (LDA) score > 3 were displayed. CON group, control group lambs; MP group, microplastic-exposed lambs.

At the genus level, *Prevotella 1* was the most dominant bacterium in the rumen of two groups, followed by *Fibrobacter*, *Treponema 2* and *unclassified F082*. Based on the criterion that at least one sample within each group had a relative abundance ≥ 2.5%, a total of 19 predominant genera were screened in the rumen (Figure 4A). Among these genera, the relative abundance of *unclassified Muribaculaceae* in MP group was 54.35% lower than that in CON group (0.05 < *p* < 0.10), whereas no significant differences were observed for the other predominant genera between the two groups (*p* > 0.05) (Supplementary Table S4). LEfSe analysis was further performed to identify differentially abundant taxa based on the relative abundances. In the rumen, a total of 2 differential genera and 1 differential family were identified between the two groups, namely *g__Succinivibrionaceae_UCG_001*, *g__Shuttleworthia* and f__Bacteroidales_UCG_001 (Figure 4B).

### 3.5 Differences of bacterial communities in the small intestine

A total of 46 phyla were detected in the 24 digesta samples from the small intestine (jejunum and ileum). The numbers of bacterial phyla identified in the jejunum and ileum were 40 and 42 in the CON group, and 42 and 44 in the MP group, respectively. Firmicutes, Proteobacteria, Tenericutes and Actinobacteria were the predominant phyla. Based on the criterion that at least one sample within each group had a relative abundance ≥ 0.5%, 11 and 10 predominant phyla were screened in the jejunum and ileum, respectively (Table 3). In the jejunum, the relative abundance of Tenericutes in MP group was slightly lower than that in CON group (*p* = 0.087). In the ileum, the relative abundance of Tenericutes was higher in CON group than that in MP group (*p* < 0.05). Compared with CON group, the relative abundance of Proteobacteria in MP group was increased by 51.54% in the jejunum and 138.3% in the ileum (0.05 < *p* < 0.10). Additionally, the F/B ratio in the jejunum was similar between the two groups (*p* > 0.05). However, the F/B ratio in the ileum of CON group was higher than MP group (*p* < 0.05) (Figure 3B).

**Table 3:** Comparison of relative abundances (%) of representative bacterial phyla in the small intestine between the CON and MP groups.

Items	CON	MP	SEM	*p*-value
Jejunum				
Proteobacteria	37.89	57.42	10.405	0.076
Firmicutes	27.85	19.41	10.237	0.429
Tenericutes	21.52	3.31	8.664	0.087
Actinobacteria	3.34	5.78	2.780	0.399
Bacteroidetes	2.35	4.40	1.643	0.241
Kiritimatiellaeota	1.45	2.54	1.641	0.521
Epsilonbacteraeota	0.21	0.86	0.613	0.338
Patescibacteria	0.26	0.19	0.112	0.531
Acidobacteria	0.13	0.31	0.111	0.156
Chloroflexi	0.12	0.30	0.116	0.149
Lentisphaerae	0.18	0.02	0.145	0.317
Ileum				
Firmicutes	43.77	31.76	8.993	0.211
Proteobacteria	15.14	36.08	11.008	0.086
Tenericutes	18.50	9.18	3.855	0.036
Actinobacteria	6.38	4.41	2.697	0.484
Bacteroidetes	2.32	4.68	1.242	0.112
Kiritimatiellaeota	1.69	1.56	0.515	0.809
Epsilonbacteraeota	0.15	1.84	1.141	0.197
Patescibacteria	0.50	0.49	0.234	0.948
Planctomycetes	0.09	0.85	0.607	0.268
Chloroflexi	0.05	0.61	0.421	0.240

CON, control group lambs (*n* = 6); MP, microplastic-exposed lambs (*n* = 6); SEM, standard error of the mean.

**Figure 5:**
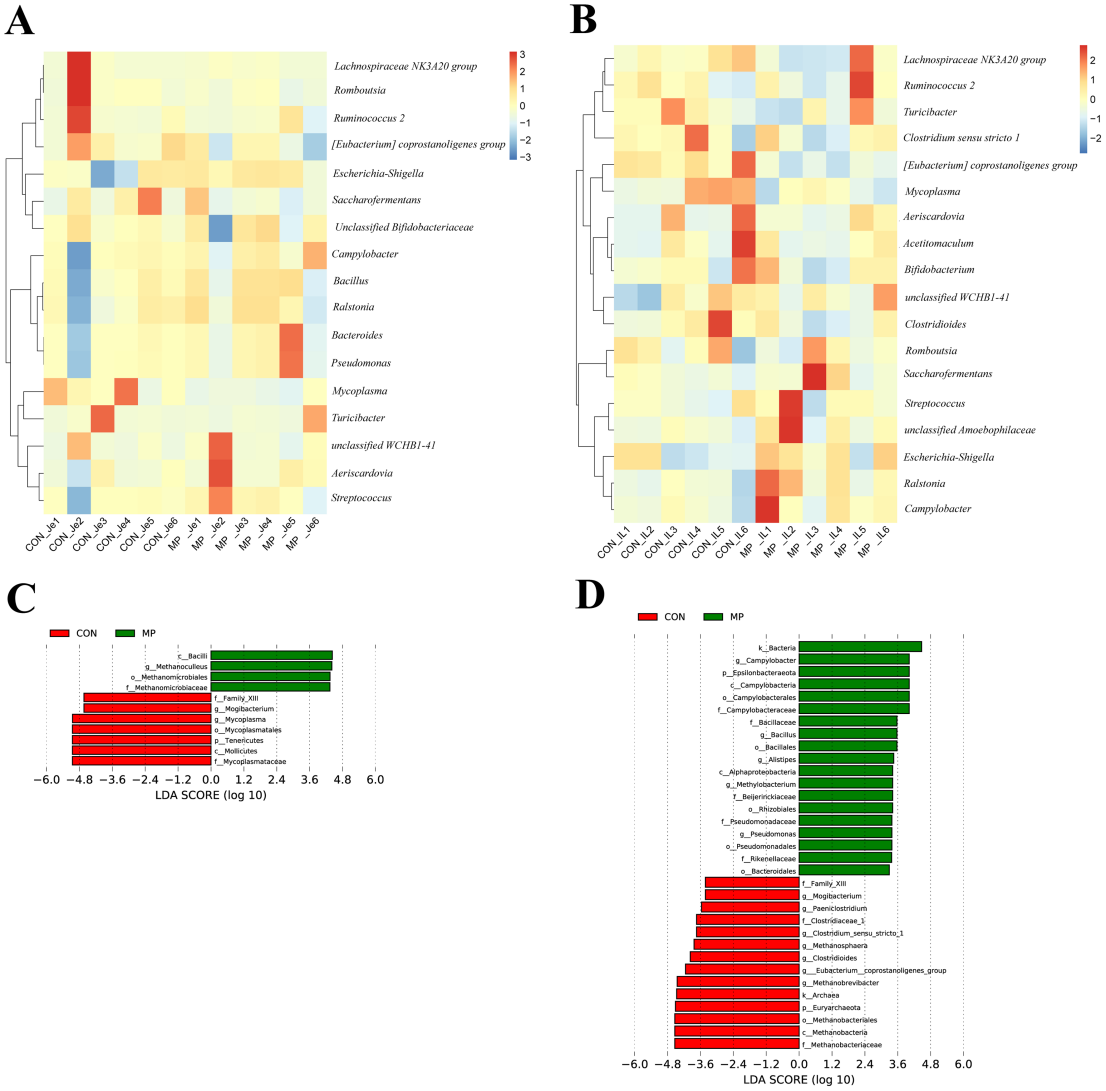
Taxonomic composition of small intestinal bacteria in the CON (*n* = 6) and MP (*n* = 6) groups. **(A)** Heatmap showing the relative abundances of predominant bacterial genera at the genus level in the jejunum. **(B)** Heatmap showing the relative abundances of predominant bacterial genera at the genus level in the ileum. Genera with a relative abundance ≥ 2.5% in at least one sample within a group were included for display. **(C)** Linear discriminant analysis effect size (LEfSe) analysis identified the most differentially abundant genera between the CON (red) and MP (green) groups in the jejunum. **(D)** LEfSe analysis identified the most differentially abundant genera between the CON (red) and MP (green) groups in the ileum. Genera with a linear discriminant analysis (LDA) score > 3 were displayed. CON group, control group lambs; MP group, microplastic-exposed lambs.

At the genus level, the predominant genera in the small intestine of lambs primarily included *Escherichia-Shigella*, *Mycoplasma* and *Turicibacter*, but their relative abundances differed between the jejunum and ileum. Based on the criterion that at least one sample within each group had a relative abundance ≥ 2.5%, 17 and 18 predominant genera were screened in the jejunum (Figure 5A) and ileum (Figure 5B), respectively. In the jejunum, compared with CON group, the relative abundance of *Mycoplasma* in MP group was decreased by 85.52%, whereas that of *Bacillus* was increased by 124.15% (0.05 < *p* < 0.10). No significant differences were observed in the other predominant genera between the two groups (*p* > 0.05) (Supplementary Table S5). In the ileum, the relative abundances of *Mycoplasma* and *[Eubacterium] coprostanoligenes group* were significantly higher in CON group than those in MP group (*p* < 0.05). Compared with CON group, the relative abundance of *Ralstonia* in the MP group was increased by 428.10%, whereas that of *Clostridioides* was decreased by 81.97% (0.05 < *p* < 0.10) (Supplementary Table S5). In the jejunum and ileum, 3 and 12 differentially abundant bacteria were identified between the two groups, respectively (Figures 5C,D). Among them, the *Mycoplasma* and *Methanobrevibacter* exhibited the most significant differences between the two groups in their respective intestinal segments.

### 3.6 Differences of bacterial communities in the large intestine

A total of 24 phyla were identified in the 24 digesta samples from the large intestine. In the cecum and colon, 20 and 20 phyla were identified in the CON group, and 23 and 21 phyla were identified in the MP group, respectively. The majority of bacteria belonged to Firmicutes (cecum: 43.18% in CON vs. 46.34% in MP; colon: 44.05% in CON vs. 49.69% in MP), Bacteroidetes (cecum: 44.29% in CON vs. 42.29% in MP; colon: 42.30% in CON vs. 39.88% in MP) and Proteobacteria (cecum: 3.77% in CON vs. 2.26% in MP; colon: 3.83% in CON vs. 2.20% in MP), which collectively accounted for approximately 90% of all bacterial taxa. Based on the criterion that at least one sample within each group had a relative abundance ≥ 0.5%, 10 and 13 predominant phyla were screened in the cecum and colon, respectively (Table 4). In the cecum, the relative abundance of Proteobacteria was higher in CON group than that in MP group (*p* < 0.05), whereas no significant differences were observed for the other phyla between the two groups (*p* > 0.05) (Table 4). In the colon, the relative abundances of Firmicutes and Elusimicrobia were lower in CON group than those in MP group (*p* < 0.05), while the relative abundance of Proteobacteria in MP group was decreased by 42.68% as compared with CON group (0.05 < *p* < 0.10) (Table 4). Additionally, the F/B ratio did not differ significantly between the two groups in the cecum (*p* > 0.05), but was significantly different in the colon (*p* < 0.05) (Figure 3C).

**Table 4:** Comparison of relative abundances (%) of representative bacterial phyla in the large intestine between the CON and MP groups.

Items	CON	MP	SEM	*p*-value
Cecum				
Firmicutes	43.18	46.34	2.715	0.272
Bacteroidetes	44.29	42.29	3.075	0.531
Proteobacteria	3.77	2.26	0.615	0.034
Epsilonbacteraeota	2.70	1.46	1.272	0.353
Spirochaetes	1.88	1.66	0.864	0.797
Tenericutes	1.64	1.44	0.298	0.530
Fibrobacteres	0.34	1.06	0.447	0.140
Verrucomicrobia	0.51	0.61	0.214	0.641
Planctomycetes	0.35	0.26	0.272	0.766
Lentisphaerae	0.18	0.42	0.237	0.320
Colon				
Firmicutes	44.05	49.69	1.517	0.007
Bacteroidetes	42.30	39.88	1.709	0.187
Proteobacteria	3.83	2.20	0.691	0.056
Epsilonbacteraeota	4.15	1.50	2.210	0.279
Tenericutes	1.78	1.36	0.273	0.174
Spirochaetes	1.54	1.54	0.835	0.996
Fibrobacteres	0.37	0.97	0.425	0.182
Verrucomicrobia	0.56	0.76	0.236	0.407
Lentisphaerae	0.36	0.18	0.276	0.516
Planctomycetes	0.28	0.23	0.235	0.833
Kiritimatiellaeota	0.14	0.23	0.100	0.387
Elusimicrobia	0.02	0.34	0.089	0.015
Unclassified phylum	0.02	0.11	0.089	0.354

CON, control group lambs (*n* = 6); MP, microplastic-exposed lambs (*n* = 6); SEM, standard error of the mean.

**Figure 6:**
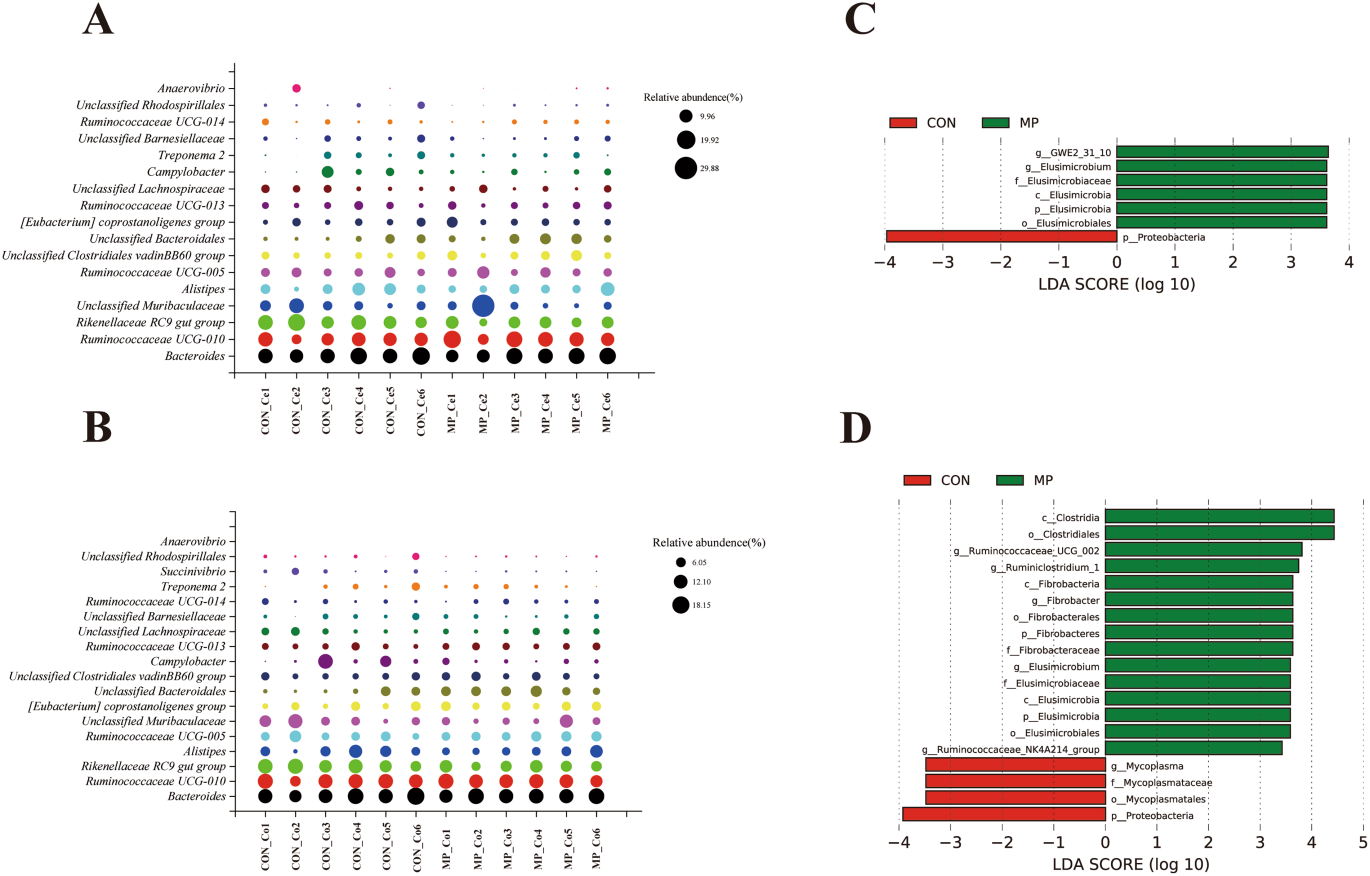
Taxonomic composition of large intestinal bacteria in the CON (*n* = 6) and MP (*n* = 6) groups. **(A)** Bubble chart showing the relative abundances of predominant bacterial genera at the genus level in the cecum. **(B)** Bubble chart showing the relative abundances of predominant bacterial genera at the genus level in the colon. Genera with a relative abundance ≥ 2.5% in at least one sample within a group were included for display. **(C)** Linear discriminant analysis effect size (LEfSe) analysis identified the most differentially abundant genera between the CON (red) and MP (green) groups in the cecum. **(D)** LEfSe analysis identified the most differentially abundant genera between the CON (red) and MP (green) groups in the colon. Genera with a linear discriminant analysis (LDA) score > 3 were displayed. CON group: Control group lambs; MP group: Microplastic-exposed lambs.

At the genus level, the *Bacteroides* was the predominant genus in the large intestine of CON and MP groups, followed by *Ruminococcaceae UCG-010*, *Rikenellaceae RC9 gut group*, *unclassified Muribaculaceae* and *Alistipes*. Based on the criterion that at least one sample within each group had a relative abundance ≥ 2.5%, 17 and 18 predominant genera were screened in the cecum (Figure 6A) and colon (Figure 6B), respectively. In the cecum, the relative abundance of *Rikenellaceae RC9 gut group* was higher in CON group than that in MP group (*p* < 0.05), whereas no significant differences were observed for the other genera between the two groups (*p* > 0.05) (Supplementary Table S6). In the colon, compared with MP group, the relative abundance of *unclassified Bacteroidales* was significantly reduced in CON group, while the relative abundance of *Succinivibrio* was significantly increased (*p* < 0.05) (Supplementary Table S6). In the cecum and colon, 2 and 6 differentially abundant bacteria were identified between the two groups, respectively (Figures 6C,D). Among them, the *GWE2_31_10* and R*uminococcaceae UCG-002* exhibited the most significant differences between the two groups in their respective intestinal segments.

### 3.7 Prediction of molecular functions of bacterial communities using PICRUSt2

In the first level of predictions, a total of 6 gene functional categories were identified, including genetic information processing (36.26 ± 0.479%), metabolism (16.03 ± 0.057%), cellular processes (15.10 ± 0.160%), human diseases (11.71 ± 0.178%), organismal systems (11.81 ± 0.105%) and environmental information processing (9.09 ± 0.254%) (Supplementary Table S7). In CON group, cellular processes did not differ significantly among gastrointestinal regions (*p* > 0.05), whereas significant differences were observed across gastrointestinal regions in MP group (*p* < 0.05). All other functional categories differed significantly among gastrointestinal regions within each group (*p* < 0.05). In the rumen, no significant differences were found between the two groups for any of the six gene functional categories (*p* > 0.05). The human diseases-related gene functional category differed significantly between the two groups in the large intestine (*p* < 0.05), and showed a significant trend in the small intestine (0.05 < *p* < 0.10). Cellular processes in the jejunum, as well as genetic information processing in the jejunum and ileum, showed significant trends between the two groups (0.05 < *p* < 0.10), and besides, organismal systems in the colon also showed a significant trend between the two groups (0.05 < *p* < 0.10) (Supplementary Table S7).

At the second level, a total of 46 gene families were identified across all digesta samples, of which 7 gene families corresponded to the major functional categories (relative abundance ≥ 4.5% in at least one gastrointestinal region within either group). These gene families were aging, cell motility, antineoplastic drug resistance, folding, sorting and degradation, nucleotide metabolism, replication and repair, and translation (Supplementary Table S8). In the rumen, the relative abundances of replication and repair in the MP group were slightly higher than those in the CON group (*p* = 0.073), whereas no significant differences were observed for the other gene families (*p* > 0.05). In the small intestine, the aging-related gene family was increased by 9.90% in MP group as compared with CON group in the jejunum (*p* = 0.065) and by 12.13% in the ileum (*p* = 0.086). Also, the replication and repair and translation were slightly higher in CON group than those in MP group (0.05 < *p* < 0.10). Nucleotide metabolism was also slightly higher in CON group than MP group in the ileum (0.05 < *p* < 0.10). Folding, sorting and degradation differed significantly between the two groups (*p* < 0.05). In the large intestine, nucleotide metabolism and replication and repair were slightly higher in MP group than those in CON group in the cecum (0.05 < *p* < 0.10), but were significantly higher in the colon (*p* < 0.05). Moreover, translation was higher in MP group than CON group in both the cecum and colon (*p* < 0.05) (Supplementary Table S8).

**Figure 7:**
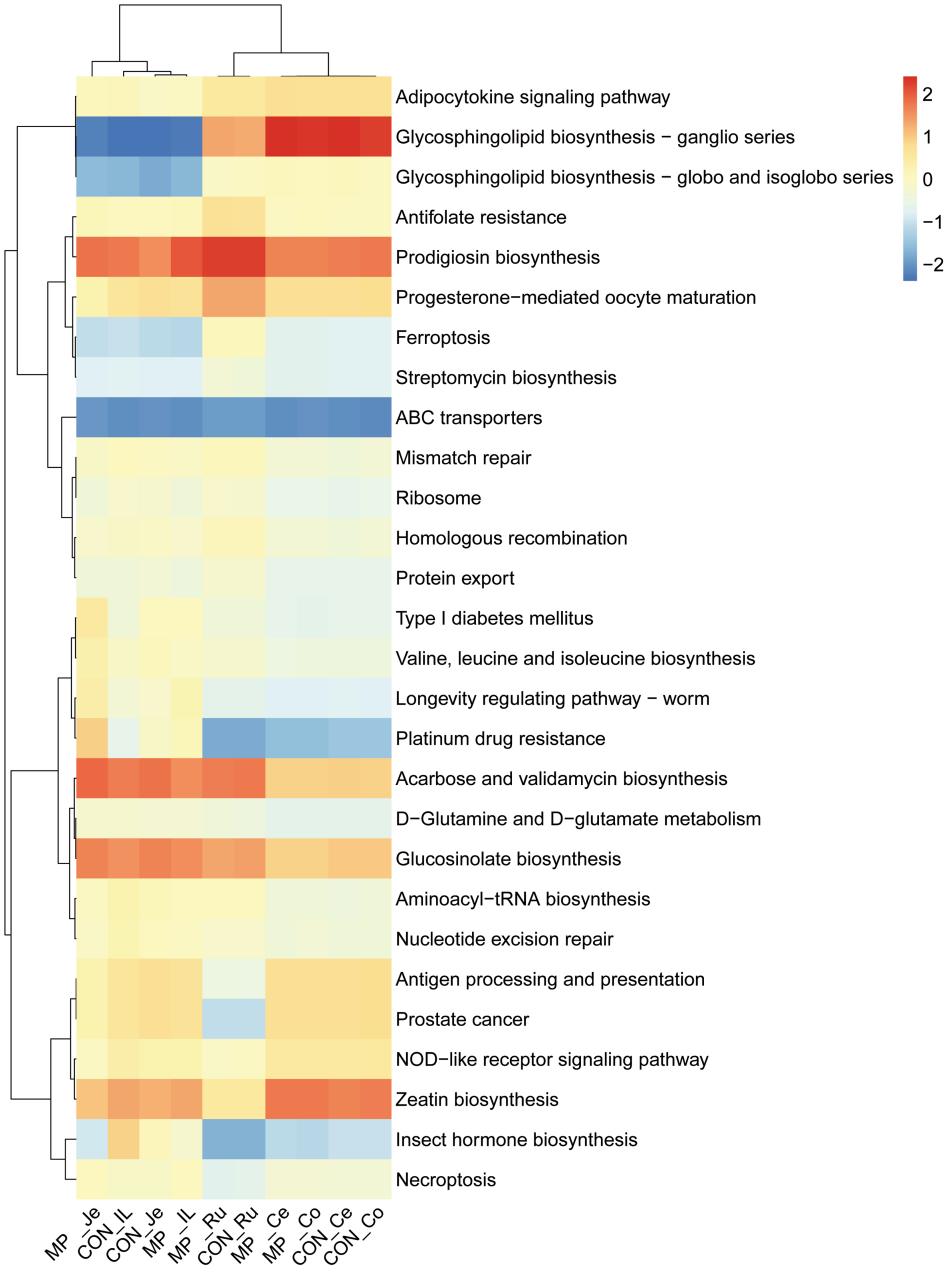
Heatmap of relative abundances of third-level KEGG ortholog (KO) functional pathways across gastrointestinal regions in the CON and MP groups (pathways with an average relative abundance ≥ 1% in at least one region were displayed). CON group, control group lambs; MP group, microplastic-exposed lambs.

At the third level, a total of 28 KO pathways were screened from 389 total pathways (average relative abundance ≥ 1% in at least one region within either group). The relative abundances of these pathways were evaluated using a Pearson distance matrix and visualized as a heatmap (Figure 7). The results showed that regardless of group, the rumen pathways were clearly separated from those of other intestinal regions and clustered independently, whereas the small intestine (jejunum and ileum) clustered together with similar abundance patterns, and the large intestine (cecum and colon) formed another distinct cluster with similar abundance patterns. The overall pathway abundance profiles were similar between the MP and CON groups within the same gastrointestinal region.

## 4 Discussion

The gastrointestinal tract harbors a vast array of microbial communities that engage in mutualistic and regulatory interactions with the host, thereby maintaining the long-term stability of the gut microbial environment and playing a critical role in the health and productivity of animals. The stability of gastrointestinal microbiota is attributed to the inter-community interactions and the inherent plasticity of communities themselves (Li et al., 2022). However, some external environmental factors, such as microplastics, can disrupt the gut environment and alter the composition and function of microbial communities (Litvak et al., 2017). As an emerging environmental contaminant, the effects of microplastics on gut microbiota represent a frontier interdisciplinary field spanning environmental toxicology and animal nutrition. To date, studies have demonstrated that microplastics can affect gastrointestinal microorganisms, but most of researches have focused on aquatic animals and rodents (Zhou et al., 2026; Magdy et al., 2026). Nevertheless, given the ubiquitous nature of microplastics, ruminants are inevitably exposed to microplastics over the long term. Unfortunately, the effects of microplastic exposure on the gastrointestinal microbiota of ruminants remain largely unexplored. Hence, the present study used fattening Hu sheep lambs as experimental models to compare the microbial community compositions of the rumen, jejunum, ileum, cecum and colon between the control (CON) and microplastic-fed (MP) groups via 16S rRNA sequencing, aiming to elucidate the unique patterns of microplastic-induced disturbance to the gastrointestinal microecology in lambs.

Alpha diversity focuses on the species composition and structural characteristics within a sample at a specific spatial or temporal scale (Kavitha et al., 2024), and it is typically quantified using species richness, Shannon index, Chao1 index and PD index. In goats, a previous study has shown that under normal conditions, the *α*-diversity of gastrointestinal bacteria was highest in the stomach, followed by the duodenum and hindgut (cecum and colon), whereas the jejunum and ileum exhibited the lowest α-diversity (Hu et al., 2024), which was consistent with the α-diversity result of CON group in the present study. However, from an overall perspective, compared with CON lambs, all diversity indexes in the rumen of MP lambs were decreased, disrupting the originally highest diversity in the rumen, while diversity in the jejunum, ileum, cecum and colon was increased. In the current study, no significant differences in α-diversity indexes were observed between the two groups in the jejunum and cecum. In the rumen, the Shannon index of MP group was slightly lower than that of CON group, whereas the other indexes did not differ significantly. The Chao1 and PD indexes in the ileum differed significantly between the two groups, and all indexes in the colon differed between CON and MP lambs. Chang et al. evaluated the α-diversity of rumen microbial communities under PS exposure (25, 50 and 100 μm) at a dose of 100 mg/d for 60 days, and results found that no significant differences in any of the indexes (Chao1, Shannon, observed species, and Faith’s PD) among treatment groups (Chang et al., 2024). This is somewhat inconsistent with the present results, which may be attributable to differences in exposure dose and duration. In addition, this finding differs from the results of microplastic study in chickens, where the diversity and richness of the gut microbial community significantly decreased, indicating high sensitivity and low tolerance to microplastics (Li et al., 2023). The results observed in this study may be explained by the fact that the rumen serves as a crucial fermentation site in the digestive system of ruminants, harboring a highly complex microbial community. Although microplastic exposure led to a declining trend in the Shannon index, suggesting structural reorganization of the ruminal microbial ecosystem, this effect may not have reached statistical significance in the short term, likely due to the inherent complexity and stability of the ruminal microbiome.

In the small intestine, the intensity of peristalsis and segmentation varies regionally, being strongest in the duodenum and jejunum to promote rapid chyme mixing and nutrient absorption (Cheng et al., 2025). Consequently, microplastics have a short residence time in the jejunum, and the jejunal microbiota are low in abundance and simple in structure. The cecum serves as the primary site for hindgut fermentation, harboring a highly stable microbial community with functional redundancy that maintains overall function and diversity stability. Therefore, these two intestinal segments possess strong buffering capacity against external disturbances, such as microplastics. The ileum, as the terminal segment of the small intestine, has reduced peristalsis, facilitating microplastic accumulation. On the other hand, microplastic particles serve as artificial substrates that promote bacterial colonization and biofilm formation. Additionally, microplastics enter the colon with the digesta and remain for a prolonged period, continuously stimulating the microbial community and inducing compositional changes. In summary, microplastics altered the normal spatial distribution pattern of highest diversity in the rumen and lowest in the small intestine, resulting in decreased rumen diversity and increased ileal and colonic diversity. The ileum is a critical site for immunity and nutrient absorption, and the increase in microbial richness induced by microplastic may pose unknown risks for host–microbe interactions. Furthermore, microplastics altered the evenness and phylogenetic composition of the colonic microbiota. The jejunum and cecum exhibited relatively strong stability, revealing that the effects of microplastics were intestinal segment-specific, likely depending on differences in microbial community structure, local fluid dynamics and plastic residence time. *β*-diversity results showed that microplastics did not cause uniform changes across all intestinal segments but preferentially disturbed the microbial communities in the rumen and ileum, and the colon showed a trend toward change, whereas the jejunum and cecum remained relatively stable. This intestinal segment-specific community response pattern reflects the differences in sensitivity to microplastic exposure in different regions of the gastrointestinal tract of lambs. A recent study has shown that short-term exposure to PS MPs significantly alters the β-diversity of the rumen microbiome in yaks, which is similar to the findings of the present study (Zhang et al., 2026).

In the gastrointestinal microbes of ruminants, the rumen harbors an extremely diverse microbial community that plays a crucial role in fermentation and occupies an important position in ruminant nutrition (Kholif et al., 2026). This is because the ruminal microbes can decompose and convert feed components, including cellulose, hemicellulose, protein, lipids and starch, into short-chain fatty acid (SCFA) and microbial protein. Under normal physiological conditions, the microbial community in the rumen maintains a dynamic equilibrium that positively contributes to the growth and health of ruminants. However, the diet, environment, age, medication and abnormal physiological states can all affect the gastrointestinal microbial community and may adversely impact gastrointestinal homeostasis (Cholewińska et al., 2021). In the current study, an unclassified phylum in CON group was higher than that in MP group. However, owing to the unclassified status, its function remains unknown, and future studies combining metagenomics and other techniques are warranted for further analysis. Muribaculaceae is a probiotic family associated with SCFA production (Zhu et al., 2024; Hu, 2023). The SCFAs play a critical role in maintaining intestinal mucosal barrier homeostasis and regulating energy metabolism (Zang et al., 2026). Our results showed that microplastics exposure led to a decreased relative abundance of *unclassified Muribaculaceae*. *Bacteroidales_UCG-001* belongs to the phylum Bacteroidetes and is typically involved in the degradation and fermentation of structural carbohydrates (e.g., cellulose and hemicellulose) (Ferreira et al., 2025), and its enrichment in MP group may be related to a compensatory enhancement of fiber substrate degradation pathways in the rumen under microplastic exposure. Furthermore, *Succinivibrionaceae_UCG-001* is an active participant in carbohydrate metabolism in the rumen and plays a key regulatory role in the propionate metabolic pathway (Castillo-Lopez et al., 2024). This taxon also participates in starch and protein degradation processes, and it was enriched in MP group. A previous study has shown that *Shuttleworthia* was highly positively correlated with propionate, total SCFA, acetate and butyrate contents (Hao et al., 2021), and besides, the persistent presence of this genus in a healthy rumen ecosystem is considered an important indicator of ruminal fermentation homeostasis. The significant enrichment of *Shuttleworthia* in CON lambs suggested that the suppression or marked reduction of *Shuttleworthia* in MP lambs, indicating that microplastic exposure may preferentially disrupt certain microbial taxa that are positively associated with rumen health. Consequently, only specific taxa with strong metabolic resilience (e.g., *Bacteroidales_UCG-001* and *Succinivibrionaceae_UCG-001*) were able to proliferate in the MP group.

After entering the rumen, microplastics are not biologically inert substances. Existing *in vitro* fermentation study has demonstrated that regardless of polymer type, dose and particle size, microplastics reduce cumulative gas production but enhance total dry matter degradation in a dose-dependent manner, and besides, microplastics interact with rumen microbes, and portions of the plastics are degraded by microbes (Eichinger et al., 2025). Under the stress conditions caused by microplastic exposure, some microbial taxa were decreased or disappeared because of maladaptation, whereas the increase of other taxa indicated that microplastic exposure enabled certain bacteria to adapt to the rumen environment. The present findings revealed that the complex disturbance mechanism of microplastics on the rumen microecology is not a simple uniform inhibition but rather an uneven impact on specific functional communities with directional replacement. Future studies should integrate multi-omics approaches, including metagenomics and metatranscriptomics, to elucidate the true functional driver networks underlying these differential microbial taxa.

The gut microbiota plays a vital role in host health by promoting intestinal development, maintaining immune function and facilitating nutrient absorption, and the balance of gut microbiota is essential for growth and development of animals (Negash, 2022). More and more evidences indicated that microplastics exposure can disrupt and impair gut microbiota function (Li et al., 2022a; Li et al., 2022a) and may even lead to occurrence of diseases (Thaiss et al., 2016). In this study, the F/B ratio in the ileum of MP lambs was lower than that of CON lambs. A previous research has confirmed that a higher intestinal F/B ratio is associated with obesity in humans (Turnbaugh et al., 2006). Furthermore, an elevated F/B ratio has been shown to be positively correlated with growth rate and feed conversion efficiency, indicating that Firmicutes and Bacteroidetes play important roles in energy metabolism and can provide more energy to the host (Yang et al., 2026). Our finding suggested that the energy metabolic pattern in the ileum of MP lambs may shift toward less efficient fermentation pathways. However, in the colon, the F/B ratio in MP lambs was higher than that in CON lamb. This increase may be accompanied by a restoration of the absolute dominance of Firmicutes, which may locally represent a restorative or compensatory adjustment. Proteobacteria is a major group of gram-negative bacteria that includes potential pathogens such as Salmonella and *Escherichia coli*. An increased abundance of Proteobacteria is generally regarded as a marker of gut dysbiosis and a potential risk of inflammation (Litvak et al., 2017). In this study, compared with the CON group, the relative abundance of Proteobacteria in the jejunum and ileum of the MP group increased by 51.54 and 138.3%, respectively, showing an upward trend, which suggested that microplastics may have the potential to induce local pro-inflammatory changes, but this result required further validation. The reason may be attributed to the selective enrichment and adsorption of Proteobacteria on the microplastic surface, as well as microplastic-induced inflammation and altered local oxygen partial pressure, which favor the competitive expansion and growth of facultative anaerobes such as Proteobacteria. In poultry, a research has reported that PS MPs exposure increased the abundance of Proteobacteria in the gut of quail (You et al., 2025), which was consistent with our results. On the contrary, the relative abundance of Proteobacteria in the colon and cecum of MP lambs was decreased as compared with CON lambs. This reversal may be explained by the fact that after microplastic particles pass from the stomach and small intestine into the large intestine, they may be partially degraded and adsorb more intestinal contents, altering their surface properties and making them less favorable for Proteobacteria colonization. The significant increase of Firmicutes abundance in the colon of the MP group may be attributed to microplastic-induced alterations in the quantity and composition of undigested carbohydrates reaching the colon, as well as the inhibition of certain digestive enzyme activities or delayed chyme emptying in the small intestine. These effects likely result in a greater influx of fermentable substrates into the colon, thereby stimulating the proliferation of Firmicutes. Tenericutes are characterized by the absence of a cell wall, and some members of this phylum in the gut are associated with host metabolic interactions and immune regulation, but a low abundance of Tenericutes is generally associated with gut homeostasis (Huang et al., 2020). The current results showed that Tenericutes abundance in the jejunum and ileum of MP group was decreased, with a particularly pronounced reduction in the ileum. This finding indicated that Tenericutes may be highly sensitive to microplastics. The absence of cell wall may render them more susceptible to the effects of microplastics, and its decreased abundance may be attributable to direct cytotoxic effects of microplastics or indirect alterations of the luminal environment.

Similar to microplastic study in other animal models (You et al., 2025), changes in the relative abundance of various gut microbial taxa were also observed in the current study. At the genus level, the impact of microplastic exposure on the microbiota exhibited pronounced intestinal segment specificity. In the small intestine, the *Mycoplasma* abundabce in the jejunum and ileum of MP group was substantially reduced, which was consistent with the characteristics of cell wall-deficient and high sensitivity to environmental toxicants. Meanwhile, the *[Eubacterium] coprostanoligenes group* abundance was significantly decreased in the ileum of MP group, suggesting that microplastics may suppress cholesterol metabolism and butyrate production. Notably, the relative abundance of *Ralstonia* was elevated in the ileum of MP group, and combined with the overall increase in Proteobacteria in the small intestine, it is indicated that microplastics may promote the proliferation of opportunistic pathogens by acting as carriers and inducing low-grade inflammation.

In the jejunum, the *Bacillus* abundance was increased compensatorily by 124.15% in MP group, which might be due to the strong tolerance conferred by its spore-forming morphology. In the large intestine, the *Bacteroides* remained the dominant genus, but the MP group caused a reduction of *Rikenellaceae RC9 gut group* (associated with cellulose degradation) abundance in the cecum and colon, indicating that microplastics suppressed a certain of fiber-degrading taxa. In the colon, the relative abundance of *unclassified Bacteroidales* was significantly elevated in MP group, whereas the *Succinivibrio* was significantly higher in CON group, suggesting a shift in the large intestinal fermentation pattern with potential impairment of propionate production pathway. In summary, after microplastic exposure, the beneficial functional taxa, including *Rikenellaceae RC9 gut group*, *Succinivibrio* and the *[Eubacterium] coprostanoligenes group*, were significantly reduced, whereas potentially harmful taxa, such as *Ralstonia*, showed an increasing trend. These alterations may disrupt the gut microbial ecological balance in lambs, adversely affecting fiber digestion, SCFA production and cholesterol metabolism. Unfortunately, these speculations have not yet been supported by direct metabolic evidence, and further studies are warranted in the future. Moreover, a previous study has shown that microplastics can disrupt gut microbial balance, leading to a relative decrease in beneficial bacteria and a relative increase in harmful bacteria (Zhang et al., 2023), which were consistent with the present findings. However, attention should be paid to marginally significant results and the taxonomic limitations at the genus level (e.g., the unclear function of *Clostridioides*). Future studies should combine absolute quantification with metabolomic validation.

Based on PICRUSt2 functional prediction of the gastrointestinal bacterial communities in fattening lambs, the most significant functional categories at the KEGG Level 2 were gene families associated with aging, cell motility, antineoplastic drug resistance, folding, sorting and degradation, nucleotide metabolism, replication and repair, and translation (Su et al., 2025). In the rumen, the replication and repair-related gene family were slightly higher in MP group than those in CON group, whereas no significant differences were observed for the other 6 core families. This finding suggested that the rumen, as the primary fermentation chamber in ruminants, may possess a certain buffering capacity and tolerance to microplastics exposure, and the elevated replication and repair-related gene family indicated that microplastics may induce low-level DNA damage stress. Overall, the rumen microecosystem may exhibit functional homeostasis under short-term microplastic exposure.

In the jejunum and ileum, the aging-related functional gene families in the jejunum and ileum of the MP group showed an increasing trend, implying that microplastics may induce cellular senescence in the small intestinal mucosa and colonizing microbes, or may be related to the local oxidative stress and chronic inflammatory microenvironment triggered by microplastics. Furthermore, the CON group showed slightly higher levels of replication and repair, translation, and nucleotide metabolism in the ileum as compared with MP group, possibly because the CON group maintained more efficient protein synthesis and genetic information processing capacity, whereas microplastic exposure may have suppressed these anabolic pathways. In the large intestine, the nucleotide metabolism and replication and repair-related gene families were slightly higher in MP group than those in CON group in the cecum, while in the colon, these two types of gene families were significantly higher than those in CON group. Meanwhile, the translation-related gene family was significantly elevated in MP group in the cecum and colon. These results indicated that microplastics promoted microbial proliferative activity, DNA replication, repair capacity and protein synthesis efficiency in the large intestine, particularly in the colon. This may be attributed to the longer retention time of microplastics in the large intestine, where they act as physical substrates or potential carbon sources to enrich specific functional microbes. Additionally, microplastics-induced mild mucosal damage may trigger compensatory hyperplastic responses in local tissues and microbial community. The functional differences among gastrointestinal regions may be closely related to variations in pH, microplastics retention time, anaerobicity, microbial community composition and host–microbe interaction patterns across segments. These findings suggested that molecular functional differences existed among intestinal regions, and microplastics may not alter the fundamental clustering pattern among regions but may affect the relative abundance of individual pathways in different intestinal segments. In this study, the failure to further validate these functional gene predictions constitutes a limitation. Future studies should further validate these functional genes and integrate functional assessments of the digestive tract epithelium and mucosal barrier, among other indicators, to determine whether microbial functional genes truly translate into deleterious or compensatory effects on mucosal homeostasis and cellular senescence. Coupling metagenomic functional predictions with host tissue responses will provide a more comprehensive evaluation of the risk mechanisms underlying microplastic exposure on the gastrointestinal health of ruminants.

## 5 Conclusion

In the current study, results showed that in the rumen, microplastics exposure led to a decreased relative abundance of *unclassified Muribaculaceae* associated with SCFA production, and the Shannon index in MP group was slightly lower than that in CON group. In the jejunum and ileum, microplastics exposure slightly increased the relative abundance of Proteobacteria, and significantly reduced the Tenericutes abundance. In contrast, microplastics exposure reduced the relative abundance of Proteobacteria in the cecum and colon, whereas Firmicutes and Elusimicrobia were elevated in the colon of MP group. Furthermore, microplastics exposure led to a slight increase in the pathogenic genus *Ralstonia* in the jejunum, while the relative abundance of the cellulose degradation-associated *Rikenellaceae RC9 gut group* was reduced in the large intestine. Taken together, polystyrene microplastics triggered a segmental and region-specific dysbiosis of the gut microbiota from the rumen to the colon in fattening lambs, with the ileum and colon being the most susceptible target segments. The present study provides a foundational reference for the differential assessment of intestinal risks associated with microplastic exposure in ruminants. Future studies should integrate metagenomics and metabolomics to elucidate the functional microbial response driven by microplastics.

## Data Availability

The sequences from the current study have been deposited in the Sequence Read Archive database of National Center for Biotechnology Information with the accession number PRJNA1470242.
