# Supplementary material for: Polystyrene microplastic stress induces adaptive responses and functional remodeling of gastrointestinal microbiota in fattening lambs
Source: Front Microbiol. 2026 Aug 5;17:1892256. doi: 10.3389/fmicb.2026.1892256 (PMC13485983; doi:10.3389/fmicb.2026.1892256)
Supplement: Supplementary file 1 [file Supplementary_file_1.docx]

Supplementary Material

# SUPPLEMENTARY TABLES

**TABLE S1 |** Feed ingredients and nutritional levels of basal diet (DM basis).

| **Ingredients, %** |  | **Nutritional levels, %** |  |
| --- | --- | --- | --- |
| Alfalfa hay | 21.80 | CP | 16.81 |
| Corn silage | 18.20 | NDF | 30.52 |
| Corn | 32.60 | ADF | 19.62 |
| Wheat bran | 6.40 | EE | 4.08 |
| Soybean meal | 9.22 | Ca | 0.82 |
| [Cottonseed](E:/%E6%9C%89%E9%81%93/Dict/8.9.4.0/resultui/html/index.html#/javascript:;) [meal](E:/%E6%9C%89%E9%81%93/Dict/8.9.4.0/resultui/html/index.html#/javascript:;) | 5.90 | P | 0.51 |
| Extruded soybean | 2.80 | ME^2^, MJ/kg | 9.20 |
| Limestone | 0.58 |  |  |
| CaHPO_4_ | 0.52 |  |  |
| NaCl | 0.48 |  |  |
| Premix^1^ | 1.50 |  |  |

DM, dry matter; CP, crude protein; NDF, neutral detergent fiber; ADF, acid detergent fiber; EE, ether extract; ME, metabolic energy.

^1^The premix supplied following per kg of diet: vitamin A 3800 IU, vitamin D_3_ 900 IU, vitamin E 30 IU, Fe 90 mg, Zn 60 mg, Mn 30 mg, Cu 8 mg, I 0.50 mg, Se 0.32 mg, Co 0.10 mg,

^2^ME was a calculated value; the other nutritional levels were measured values.

**TABLE S2 |** Data acquisition and ASV statistics of all samples.

| **Items** | **Raw tags** | **Effective tags** | **Average Length(bp)** | **ASV count** | **Q30 (%)** |
| --- | --- | --- | --- | --- | --- |
| CON_Ru1 | 57576 | 57522 | 291.7 | 1012 | 99.01 |
| CON_Ru2 | 56612 | 56571 | 291.6 | 769 | 99 |
| CON_Ru3 | 73131 | 73080 | 291.4 | 1191 | 98.92 |
| CON_Ru4 | 55233 | 55196 | 291.5 | 1004 | 98.82 |
| CON_Ru5 | 73098 | 73061 | 291.7 | 1052 | 99.04 |
| CON_Ru6 | 56221 | 56173 | 291.4 | 743 | 99 |
| MP_Ru1 | 50639 | 50601 | 291.6 | 781 | 98.95 |
| MP_Ru2 | 76054 | 75984 | 291.7 | 810 | 99.05 |
| MP_Ru3 | 57253 | 57192 | 291.5 | 876 | 99.04 |
| MP_Ru4 | 73480 | 73428 | 291.5 | 869 | 98.97 |
| MP_Ru5 | 65745 | 65687 | 291.4 | 696 | 98.89 |
| MP_Ru6 | 69576 | 69532 | 291.5 | 953 | 98.97 |
| CON_Je1 | 78984 | 78892 | 291.8 | 784 | 98.87 |
| CON_Je2 | 55391 | 55316 | 291.8 | 851 | 98.92 |
| CON_Je3 | 77135 | 77035 | 291.6 | 499 | 98.98 |
| CON_Je4 | 53535 | 53478 | 291.6 | 474 | 98.9 |
| CON_Je5 | 68362 | 68285 | 292.8 | 742 | 99.05 |
| CON_Je6 | 77068 | 76995 | 292.4 | 899 | 98.79 |
| MP_Je1 | 58499 | 58427 | 293.5 | 708 | 98.89 |
| MP_Je2 | 73933 | 73830 | 292.1 | 1040 | 98.89 |
| MP_Je3 | 67829 | 67768 | 292.1 | 734 | 98.88 |
| MP_Je4 | 59417 | 59358 | 292.1 | 607 | 98.94 |
| MP_Je5 | 51063 | 50998 | 292.9 | 1058 | 98.79 |
| MP_Je6 | 56934 | 56876 | 291.7 | 469 | 98.92 |
| CON_IL1 | 73922 | 73823 | 291.6 | 486 | 98.97 |
| CON_IL2 | 53594 | 53528 | 291.5 | 362 | 99 |
| CON_IL3 | 59403 | 59329 | 291.6 | 627 | 98.99 |
| CON_IL4 | 65004 | 64925 | 291.4 | 507 | 98.92 |
| CON_IL5 | 76448 | 76342 | 291.6 | 623 | 98.88 |
| CON_IL6 | 64319 | 64255 | 291.6 | 604 | 98.89 |
| MP_IL1 | 50241 | 50196 | 293 | 665 | 98.76 |
| MP_IL2 | 69715 | 69632 | 292 | 1329 | 98.95 |
| MP_IL3 | 54916 | 54837 | 291.9 | 522 | 99 |
| MP_IL4 | 66141 | 66044 | 293.2 | 987 | 98.92 |
| MP_IL5 | 74622 | 74542 | 291.5 | 642 | 99.03 |
| MP_IL6 | 75605 | 75515 | 291.7 | 763 | 98.99 |
| CON_Ce1 | 65556 | 65504 | 291.4 | 812 | 98.9 |
| CON_Ce2 | 51469 | 51411 | 291.6 | 492 | 98.9 |
| CON_Ce3 | 57524 | 57454 | 292 | 730 | 98.81 |
| CON_Ce4 | 65631 | 65552 | 291.7 | 725 | 98.86 |
| CON_Ce5 | 65022 | 64945 | 291.8 | 853 | 98.94 |
| CON_Ce6 | 67839 | 67752 | 291.6 | 729 | 98.85 |
| MP_Ce1 | 52760 | 52708 | 291.6 | 660 | 98.89 |
| MP_Ce2 | 61279 | 61206 | 291.2 | 613 | 98.93 |
| MP_Ce3 | 75784 | 75704 | 291.5 | 939 | 98.92 |
| MP_Ce4 | 79442 | 79359 | 291.8 | 873 | 98.87 |
| MP_Ce5 | 62301 | 62247 | 291.7 | 831 | 98.86 |
| MP_Ce6 | 59505 | 59439 | 291.5 | 729 | 98.93 |
| CON_Co1 | 78789 | 78713 | 291.4 | 883 | 98.81 |
| CON_Co2 | 60635 | 60564 | 291.5 | 546 | 98.91 |
| CON_Co3 | 75359 | 75247 | 291.6 | 830 | 98.95 |
| CON_Co4 | 56069 | 55997 | 291.5 | 699 | 98.86 |
| CON_Co5 | 56900 | 56842 | 291.6 | 799 | 98.85 |
| CON_Co6 | 65130 | 65062 | 291.5 | 712 | 98.92 |
| MP_Co1 | 61843 | 61774 | 291.6 | 728 | 98.89 |
| MP_Co2 | 66773 | 66708 | 291.4 | 1051 | 98.88 |
| MP_Co3 | 73093 | 73010 | 291.3 | 1031 | 98.88 |
| MP_Co4 | 56735 | 56670 | 291.6 | 797 | 98.89 |
| MP_Co5 | 59242 | 59165 | 291.5 | 877 | 98.9 |
| MP_Co6 | 77539 | 77441 | 291.6 | 837 | 98.89 |
| Total | 3878917 | 3874727 | / | 46514 | / |
| Mean | 64649 | 64579 | 291.8 | 775 | 98.92 |
| SE | 1133 | 1131 | 0.1 | 25 | 0.01 |

**TABLE S3 |** Analysis of permutational multivariate analysis of variance results of bacterial community according to gastrointestinal regions between the CON and MP groups.

| **Regions** | **Groups** | **R^2^** | ***p*-value** |
| --- | --- | --- | --- |
| Rumen | CON vs. MP | 0.13 | 0.037 |
| Jejunum | CON vs. MP | 0.12 | 0.190 |
| Ileum | CON vs. MP | 0.18 | 0.035 |
| Cecum | CON vs. MP | 0.10 | 0.276 |
| Colon | CON vs. MP | 0.12 | 0.086 |

**TABLE S4 |** Comparison of the relative abundance (%) of the representative bacteria at the genus level in the rumen of CON and MP groups.

| **Items** |  | **CON** | **MP** | **SEM** | ***p*-value** |
| --- | --- | --- | --- | --- | --- |
| Firmicutes | *Succiniclasticum* | 4.18 | 5.03 | 0.933 | 0.383 |
|  | *Ruminococcus 1* | 3.21 | 2.25 | 0.549 | 0.111 |
|  | *[Eubacterium] ruminantium group* | 1.25 | 1.86 | 0.370 | 0.129 |
|  | *Ruminococcus 2* | 0.83 | 2.16 | 1.445 | 0.378 |
|  | *Lachnospiraceae NK3A20 group* | 1.09 | 1.42 | 0.508 | 0.535 |
|  | *unclassified Veillonellaceae* | 0.72 | 0.86 | 0.791 | 0.860 |
|  | *Ruminococcaceae UCG-002* | 0.41 | 0.87 | 0.526 | 0.395 |
| Bacteroidetes | *Prevotella 1* | 20.17 | 22.88 | 2.980 | 0.386 |
|  | *unclassified F082* | 6.71 | 5.06 | 1.362 | 0.254 |
|  | *Rikenellaceae RC9 gut group* | 6.03 | 5.37 | 1.380 | 0.646 |
|  | *unclassified Bacteroidales RF16 group* | 3.03 | 3.40 | 1.070 | 0.738 |
|  | *unclassified Muribaculaceae* | 2.80 | 1.28 | 0.735 | 0.087 |
|  | *Prevotellaceae UCG-001* | 1.99 | 1.35 | 0.515 | 0.238 |
|  | *Prevotella 9* | 1.18 | 2.67 | 0.991 | 0.189 |
|  | *Prevotellaceae UCG-003* | 1.57 | 1.69 | 0.390 | 0.762 |
|  | *Prevotella 7* | 1.31 | 1.19 | 0.903 | 0.895 |
| Fibrobacteres | *Fibrobacter* | 8.11 | 9.95 | 1.567 | 0.267 |
| Spirochaetes | *Treponema 2* | 7.64 | 8.37 | 1.606 | 0.660 |
| Proteobacteria | *Succinivibrio* | 0.94 | 0.88 | 0.629 | 0.924 |

CON, control group lambs (n = 6); MP, microplastic-exposed lambs (n = 6); SEM, standard error of the mean.

**TABLE S5 |** Comparison of the relative abundance (%) of the representative bacteria at the genus level in the small intestine of CON and MP groups.

| **Items** |  | **CON** | **MP** | **SEM** | ***p*-value** |
| --- | --- | --- | --- | --- | --- |
| Jejunum |  |  |  |  |  |
| Firmicutes | *Turicibacter* | 9.44 | 4.31 | 8.971 | 0.580 |
|  | *Saccharofermentans* | 2.85 | 1.86 | 1.588 | 0.549 |
|  | *Ruminococcus 2* | 2.19 | 1.26 | 1.301 | 0.488 |
|  | *Bacillus* | 0.98 | 2.19 | 0.570 | 0.068 |
|  | *Lachnospiraceae NK3A20 group* | 1.63 | 0.95 | 1.115 | 0.558 |
|  | *[Eubacterium] coprostanoligenes group* | 1.55 | 0.81 | 0.572 | 0.245 |
|  | *Romboutsia* | 1.61 | 0.67 | 0.936 | 0.337 |
|  | *Streptococcus* | 0.19 | 1.07 | 0.771 | 0.303 |
| Proteobacteria | *Escherichia-Shigella* | 28.50 | 43.42 | 10.677 | 0.192 |
|  | *Ralstonia* | 2.04 | 3.51 | 1.034 | 0.186 |
|  | *Pseudomonas* | 0.19 | 1.21 | 0.917 | 0.316 |
| Actinobacteria | *Aeriscardovia* | 1.77 | 4.08 | 2.521 | 0.379 |
|  | *Unclassified Bifidobacteriaceae* | 0.52 | 0.54 | 0.512 | 0.975 |
| Tenericutes | *Mycoplasma* | 20.65 | 2.99 | 8.371 | 0.086 |
| Kiritimatiellaeota | *unclassified WCHB1-41* | 1.45 | 2.54 | 1.641 | 0.521 |
| Bacteroidetes | *Bacteroides* | 0.60 | 1.48 | 0.845 | 0.342 |
| Epsilonbacteraeota | *Campylobacter* | 0.19 | 0.82 | 0.615 | 0.348 |
| Ileum |  |  |  |  |  |
| Firmicutes | *Turicibacter* | 16.66 | 10.26 | 6.796 | 0.369 |
|  | *Romboutsia* | 3.48 | 2.62 | 1.419 | 0.561 |
|  | *Saccharofermentans* | 1.92 | 4.11 | 2.294 | 0.379 |
|  | *Lachnospiraceae NK3A20 group* | 2.97 | 1.87 | 1.060 | 0.323 |
|  | *[Eubacterium] coprostanoligenes group* | 3.55 | 1.20 | 0.512 | 0.001 |
|  | *Streptococcus* | 1.41 | 1.85 | 1.041 | 0.679 |
|  | *Ruminococcus 2* | 1.47 | 1.38 | 1.090 | 0.932 |
|  | *Acetitomaculum* | 1.43 | 0.85 | 0.584 | 0.345 |
|  | *Clostridioides* | 1.67 | 0.30 | 0.573 | 0.057 |
|  | *Clostridium sensu stricto 1* | 1.22 | 0.38 | 0.482 | 0.111 |
| Proteobacteria | *Escherichia-Shigella* | 12.91 | 28.28 | 10.457 | 0.172 |
|  | *Ralstonia* | 0.37 | 1.94 | 0.768 | 0.094 |
| Actinobacteria | *Aeriscardovia* | 3.81 | 2.69 | 2.522 | 0.667 |
|  | *Bifidobacterium* | 0.84 | 0.35 | 0.483 | 0.336 |
| Tenericutes | *Mycoplasma* | 17.20 | 8.57 | 3.785 | 0.046 |
| Kiritimatiellaeota | *unclassified WCHB1-41* | 1.69 | 1.56 | 0.515 | 0.809 |
| Epsilonbacteraeota | *Campylobacter* | 0.14 | 1.81 | 1.142 | 0.202 |
| Bacteroidetes | *Unclassified Amoebophilaceae* | 0.16 | 1.07 | 0.760 | 0.281 |

CON, control group lambs (n = 6); MP, microplastic-exposed lambs (n = 6); SEM, standard error of the mean.

**TABLE S6 |** Comparison of the relative abundance (%) of the representative bacteria at the genus level in the large intestine of CON and MP groups.

| **Items** |  | **CON** | **MP** | **SEM** | ***p*-value** |
| --- | --- | --- | --- | --- | --- |
| Cecum |  |  |  |  |  |
| Firmicutes | *Ruminococcaceae UCG-010* | 9.68 | 12.54 | 1.853 | 0.154 |
|  | *Ruminococcaceae UCG-005* | 4.52 | 5.00 | 1.093 | 0.670 |
|  | *Unclassified Clostridiales vadinBB60 group* | 2.88 | 4.19 | 0.820 | 0.142 |
|  | *[Eubacterium] coprostanoligenes group* | 2.85 | 3.18 | 0.881 | 0.722 |
|  | *Ruminococcaceae UCG-013* | 2.67 | 2.64 | 0.619 | 0.964 |
|  | *Unclassified Lachnospiraceae* | 2.34 | 2.05 | 0.760 | 0.711 |
|  | *Ruminococcaceae UCG-014* | 1.19 | 0.91 | 0.398 | 0.489 |
|  | *Anaerovibrio* | 0.69 | 0.10 | 0.648 | 0.406 |
| Bacteroidetes | *Bacteroides* | 13.56 | 12.78 | 1.766 | 0.669 |
|  | *Rikenellaceae RC9 gut group* | 11.23 | 7.17 | 1.688 | 0.037 |
|  | *Unclassified Muribaculaceae* | 5.83 | 7.43 | 4.750 | 0.743 |
|  | *Alistipes* | 5.83 | 5.33 | 1.701 | 0.776 |
|  | *Unclassified Bacteroidales* | 2.39 | 4.22 | 1.175 | 0.151 |
|  | *Unclassified Barnesiellaceae* | 1.53 | 0.97 | 0.572 | 0.345 |
| Epsilonbacteraeota | *Campylobacter* | 2.58 | 1.42 | 1.306 | 0.397 |
| Spirochaetes | *Treponema 2* | 1.65 | 1.08 | 0.697 | 0.436 |
| Proteobacteria | *Unclassified Rhodospirillales* | 0.94 | 0.30 | 0.505 | 0.236 |
| Colon |  |  |  |  |  |
| Firmicutes | *Ruminococcaceae UCG-010* | 11.53 | 11.19 | 1.382 | 0.810 |
|  | *Ruminococcaceae UCG-005* | 4.93 | 5.45 | 0.846 | 0.555 |
|  | *[Eubacterium] coprostanoligenes group* | 3.46 | 4.33 | 0.845 | 0.327 |
|  | *Unclassified Clostridiales vadinBB60 group* | 2.90 | 3.46 | 0.671 | 0.420 |
|  | *Ruminococcaceae UCG-013* | 2.40 | 2.88 | 0.417 | 0.278 |
|  | *Unclassified Lachnospiraceae* | 2.33 | 2.08 | 0.653 | 0.710 |
|  | *Ruminococcaceae UCG-014* | 1.24 | 1.17 | 0.440 | 0.877 |
|  | *Anaerovibrio* | 0.48 | 0.10 | 0.448 | 0.426 |
| Bacteroidetes | *Bacteroides* | 12.79 | 13.14 | 1.595 | 0.833 |
|  | *Rikenellaceae RC9 gut group* | 10.57 | 7.50 | 1.415 | 0.055 |
|  | *Alistipes* | 5.84 | 5.05 | 1.581 | 0.630 |
|  | *Unclassified Muribaculaceae* | 5.70 | 4.11 | 1.938 | 0.431 |
|  | *Unclassified Bacteroidales* | 2.37 | 5.19 | 1.061 | 0.024 |
|  | *Unclassified Barnesiellaceae* | 1.38 | 1.13 | 0.484 | 0.616 |
| Proteobacteria | *Succinivibrio* | 1.19 | 0.20 | 0.401 | 0.033 |
|  | *Unclassified Rhodospirillales* | 0.95 | 0.22 | 0.483 | 0.161 |
| Epsilonbacteraeota | *Campylobacter* | 4.03 | 1.31 | 2.217 | 0.272 |
| Spirochaetes | *Treponema 2* | 1.37 | 1.05 | 0.692 | 0.652 |

CON, control group lambs (n = 6); MP, microplastic-exposed lambs (n = 6); SEM, standard error of the mean.

**TABLE S7 |** Predicted functions on the first level of the digesta-associated bacteria throughout the gastrointestinal tract of CON and MP groups.

| **Items** | **Groups** | **Ru** | **Je** | **Il** | **Ce** | **Co** | ***p*-value** |
| --- | --- | --- | --- | --- | --- | --- | --- |
| Cellular Processes | CON | 14.21±0.094 | 15.15±0.643 | 14.25±0.41 | 15.15±0.185 | 15.23±0.153 | 0.113 |
|  | MP | 13.93±0.176a | 16.92±0.644a | 15.65±0.765ab | 15.19±0.133bc | 15.29±0.119bc | 0.003 |
| Environmental Information Processing | CON | 7.89±0.15c | 11.05±0.56a | 9.83±0.429b | 7.75±0.106c | 7.76±0.085c | ＜0.001 |
|  | MP | 7.69±0.146c | 12.45±0.681a | 11±0.778b | 7.69±0.083c | 7.78±0.08c | ＜0.001 |
| Genetic Information Processing | CON | 39.87±0.12a | 34.23±1.438c | 37.02±1.064b | 36.85±0.221b | 36.9±0.205b | 0.002 |
|  | MP | 40.24±0.192a | 30.02±1.67b | 32.97±1.915b | 37.29±0.184a | 37.2±0.134a | ＜0.001 |
| Human Diseases | CON | 9.73±0.045c | 12.8±0.353a | 11.94±0.247b | 11.66±0.078b | 11.64±0.034b | ＜0.001 |
|  | MP | 9.76±0.047c | 13.79±0.412a | 13.14±0.5a | 11.35±0.048b | 11.33±0.045b | ＜0.001 |
| Metabolism | CON | 16.72±0.095a | 15.87±0.145b | 15.68±0.101b | 15.89±0.077b | 15.8±0.047b | ＜0.001 |
|  | MP | 16.79±0.105a | 16.1±0.095b | 15.74±0.157c | 15.88±0.057bc | 15.86±0.023bc | ＜0.001 |
| Organismal Systems | CON | 11.59±0.062b | 10.91±0.224c | 11.28±0.089b | 12.69±0.074a | 12.66±0.047a | ＜0.001 |
|  | MP | 11.59±0.053b | 10.72±0.277c | 11.5±0.348b | 12.59±0.031a | 12.55±0.029a | ＜0.001 |

CON, control group lambs (n = 6); MP, microplastic-exposed lambs (n = 6); SEM, standard error of the mean.

**TABLE S8 |** Predicted functions on the second level of the digesta-associated bacteria throughout the gastrointestinal tract of CON and MP groups (relative abundance ≥ 4.5% in at least one gastrointestinal region within either group).

|  | **Groups** | **Aging** | ***p* -value** | **Cell motility** | ***p* -value** | **antineoplastic drug resistance** | ***p* -value** | **Folding, sorting and degradation** | ***p* -value** | **Nucleotide metabolism** | ***p* -value** | **Replication and repair** | ***p* -value** | **Translation** | ***p*-value** |
| --- | --- | --- | --- | --- | --- | --- | --- | --- | --- | --- | --- | --- | --- | --- | --- |
| Ruman | CON | 5.67±0.068 | 0.568 | 3.27±0.083 | 0.209 | 6.21±0.063 | 0.307 | 5.18±0.017 | 0.745 | 4.42±0.022 | 0.223 | 7.34±0.022 | 0.073 | 6.35±0.021 | 0.104 |
|  | MPs | 5.73±0.088 |  | 3.01±0.178 |  | 6.31±0.064 |  | 5.19±0.010 |  | 4.46±0.028 |  | 7.43±0.043 |  | 6.43±0.035 |  |
| Jejunum | CON | 5.42±0.201 | 0.065 | 3.16±0.456 | 0.128 | 6.38±0.064 | 0.486 | 4.61±0.086 | 0.021 | 4.24±0.098 | 0.132 | 5.93±0.319 | 0.080 | 5.57±0.284 | 0.094 |
|  | MPs | 5.95±0.162 |  | 4.14±0.375 |  | 6.46±0.101 |  | 4.21±0.117 |  | 4±0.106 |  | 5.11±0.275 |  | 4.73±0.354 |  |
| Ileum | CON | 5.42±0.099 | 0.086 | 2.75±0.291 | 0.197 | 6.41±0.127 | 0.541 | 4.75±0.074 | 0.019 | 4.34±0.057 | 0.084 | 6.4±0.198 | 0.089 | 6.15±0.211 | 0.060 |
|  | MPs | 6.08±0.331 |  | 3.51±0.467 |  | 6.52±0.138 |  | 4.4±0.100 |  | 4.05±0.131 |  | 5.64±0.354 |  | 5.21±0.388 |  |
| Cecom | CON | 5.21±0.051 | 0.493 | 3.95±0.176 | 0.802 | 6.62±0.035 | 0.298 | 4.89±0.037 | 0.207 | 4.03±0.031 | 0.089 | 6.44±0.035 | 0.053 | 5.8±0.056 | 0.048 |
|  | MPs | 5.14±0.089 |  | 4.01±0.128 |  | 6.56±0.049 |  | 4.95±0.026 |  | 4.1±0.020 |  | 6.53±0.029 |  | 5.96±0.039 |  |
| Colon | CON | 5.21±0.033 | 0.154 | 4.07±0.160 | 0.892 | 6.59±0.033 | 0.671 | 4.9±0.027 | 0.109 | 4.02±0.031 | 0.021 | 6.46±0.031 | 0.024 | 5.83±0.046 | 0.037 |
|  | MPs | 5.14±0.019 |  | 4.1±0.074 |  | 6.57±0.04 |  | 4.95±0.007 |  | 4.12±0.017 |  | 6.55±0.018 |  | 5.95±0.018 |  |

CON, control group lambs (n = 6); MP, microplastic-exposed lambs (n = 6); SEM, standard error of the mean.

# SUPPLEMENTARY FIGURES

**Supplementary Figure S1 |** Venn diagrams showing the distribution of shared and unique ASVs in the rumen (A), jejunum (B), ileum (C), cecum (D) and colon (E) between the CON and MP groups.
